# Penicibrocazines A–E, Five New Sulfide Diketopiperazines from the Marine-Derived Endophytic Fungus *Penicillium brocae*

**DOI:** 10.3390/md13010276

**Published:** 2015-01-07

**Authors:** Ling-Hong Meng, Peng Zhang, Xiao-Ming Li, Bin-Gui Wang

**Affiliations:** 1 Key Laboratory of Experimental Marine Biology, Institute of Oceanology, Chinese Academy of Sciences, Nanhai Road 7, Qingdao 266071, China; E-Mails: m8545303@163.com (L.-H.M.); zp52715@126.com (P.Z.); lixmqd@aliyun.com (X.-M.L.); 2 College of Earth Science, University of Chinese Academy of Sciences, Yuquan Road 19A, Beijing 100049, China

**Keywords:** mangrove plant, endophytic fungus, *Pencillium brocae*, secondary metabolites, antimicrobial activity

## Abstract

Five new sulfide diketopiperazine derivatives, namely, penicibrocazines A–E (**1**–**5**), along with a known congener (**6**), were isolated and identified from the culture extract of *Penicillium brocae* MA-231, an endophytic fungus obtained from the fresh tissue of the marine mangrove plant *Avicennia marina.* The structures of these compounds were elucidated by detailed interpretation of NMR and mass spectroscopic data and the structures of compounds **1** and **3** were confirmed by single-crystal X-ray diffraction analysis. All these compounds were examined for cytotoxic and antimicrobial activities. Compounds **2**–**6** exhibited antimicrobial activity against some of the tested strains with MIC values ranging from 0.25 to 64 μg/mL.

## 1. Introduction

**Figure 1:**
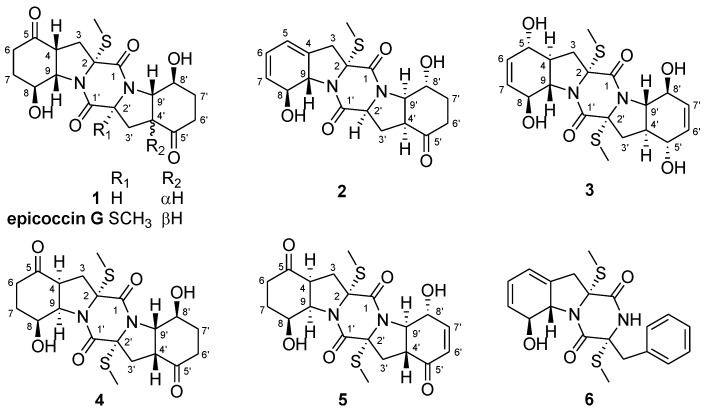
Structures of the isolated compounds **1**–**6** and reference compound epicoccin G.

Thiodiketopiperazine derivatives, which generally possess a 6-5-6-5-6 diketopiperazine skeleton with a disulfide bridge or *S*-methyl group(s), have attracted considerable attention due to their diversified structures and potent biological activities [1,2,3]. As part of our recently initiated program aimed at the discovery of bioactive secondary metabolites from marine-derived fungi, a series of structurally interesting and biologically active natural products has been described [4,5,6,7,8,9]. Very recently, six new cytotoxic bisthiodiketopiperazine derivatives, brocazines A–F, have been isolated from the culture extract of *Penicillium brocae* MA-231, an endophytic fungus obtained from the fresh tissue of the marine mangrove plant *Avicennia marina* [10]. Further work on the remaining fractions of the fungus resulted in the isolation of five new sulfide diketopiperazine derivatives, namely, penicibrocazines A–E (**1**–**5**) and one known analog (**6**) (Figure 1). The structures of these compounds were determined by detailed analysis of the NMR and mass spectrometric data and compounds **1** and **3** were confirmed by single-crystal X-ray diffraction analysis. All these compounds were examined for cytotoxic and antimicrobial activities. Details of the isolation, structure elucidation, and biological activity of compounds **1**–**6** are reported herein.

## 2. Results and Discussion

### 2.1. Structure Elucidation of the New Compounds

**Figure 2:**
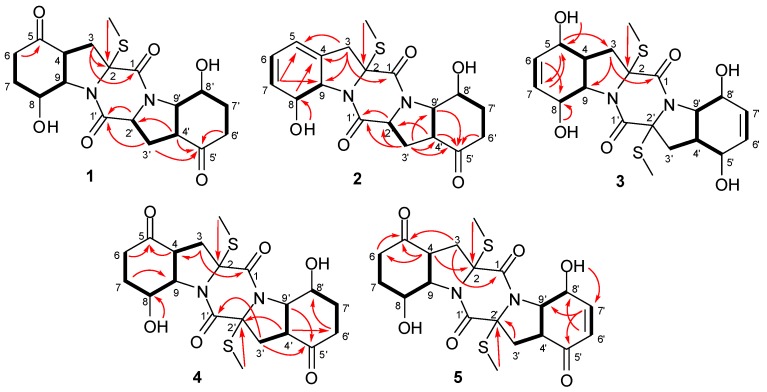
Key ^1^H-^1^H COSY (**bold lines**) and HMBC (**red arrows**) correlations of compounds **1**–**5**.

Compound **1** was obtained as colorless crystals. Its molecular formula was determined as C_19_H_2__4_N_2_O_6_S on the basis of HRESIMS, implying nine degrees of unsaturation. Detailed analyses of the ^1^H and ^13^C NMR data (Table 1 and Table 2, Supplementary Figures S1 and S2) indicated the presence of one methyl, six methylenes, seven sp^3^ methines, and five quaternary carbons (including two ketones and two ester/amide carbonyl carbons). The NMR data of **1** revealed some structural similarities to epicoccin G, a disulfide diketopiperazine isolated from the fungus *Epicoccum nigrum* [2,11]. However, the signal for a quaternary carbon resonating at δ_C_ 71.6 (C-2') in epicoccin G was absent in the ^13^C NMR spectrum of **1**. Instead, resonances for a methine group at δ_H_ 4.57 (H-2') and δ_C_ 59.6 (C-2') were observed in the NMR spectra of **1** (Table 1 and Table 2). Moreover, signals for one of the two *S-*methyl groups (δ_H_ 1.90/δ_C_ 14.2, CH_3_) in epicoccin G [2] was absent in the NMR spectra of **1**, indicating the lack of a *S*-methyl group in **1**. This deduction was confirmed by the ^1^H-^1^H COSY correlations from H_2_-3' to H-2' and H-4' as well as by the observed HMBC correlations from H-2' and H-3' to C-1' and from H-4' to C-2', as shown in Figure 2.

**Table 1:** ^1^H NMR (500 MHz) data of compounds **1**–**5** (δ in ppm, *J* in Hz).

Position	1 ^a^	2 ^b^	3 ^b^	4 ^b^	5 ^c^
3α	2.38, m (overlap)	2.89, d (16.0)	2.48, m (overlap)	2.28, m (overlap)	2.63, m (overlap)
3β	2.32, m (overlap)	3.08, d (16.0)	2.08, t (12.7)	2.22, m (overlap)	3.07, d (13.8)
4	3.30, dd (8.7, 8.0)		2.30, m	2.96, t (8.0)	3.14, t (8.6)
5		5.93, d (2.3)	4.08, d (8.8)		
6	2.42, m (overlap)	5.89, dd (9.6, 2.3)	5.55,d (10.0)	α 2.77, dd (13.3, 7.3) β 2.27, m (overlap)	2.33, m (overlap)
7	α 2.40, m (overlap) β 1.84, td (12.2, 6.2)	5.59, d (9.6)	5.69, d (10.0)	1.90, m	2.00, m
8	4.15, dd (13.1, 6.2)	4.53, d (13.4)	4.16, d (8.2)	4.33, brs	4.27, brs
9	3.57, dd (13.1, 8.0)	4.69, d (13.4)	3.43, dd (8.2, 11.9)	4.29, d (8.0)	4.51, br m
2'	4.57, dd (10.4, 7.1)	4.45, dd (10.3, 7.0)			
3'α	2.02, m (overlap)	2.56, m (overlap)	2.48, m (overlap)	2.21, m (overlap)	2.33, m (overlap)
3'β	2.92, dd (13.1, 7.1)	2.04, ddd (12.4, 10.3, 8.3)	2.08, t (12.7)	2.28, m (overlap)	2.53, dd (12.9, 4.5)
4'	3.24, dd (12.8, 6.7)	3.12, m (overlap)	2.30, m	3.40, m	3.47, td (13.0, 4.5)
5'			4.08, d (8.8)		
6'	α 2.36, m (overlap) β 2.56, ddd (17.5, 6.3, 1.8)	α 2.60, m (overlap) β 2.28, dt (16.4, 5.6)	5.55, d (10.0)	α 2.59, ddd (18.2, 12.4, 5.2) β 2.27, m (overlap)	6.01, d (10.0)
7'	α 2.12, m β 2.04, m (overlap)	α 1.74, m β 1.95, dt (12.5, 5.6)	5.69, d (10.0)	α 1.58, m β 2.27, m (overlap)	6.86, d (10.0)
8'	3.66, m	4.36, m (overlap)	4.16, d (8.2)	4.00, dd (13.2, 7.4)	4.60, d (8.2)
9'	4.45, dd (12.8, 8.2)	4.36, m (overlap)	3.43, dd (8.2, 11.9)	3.65,dd (13.2, 8.8)	3.86, dd (13.0, 8.2)
2-SMe	2.09, s	2.10, s	2.15, s	1.93, s	2.09, s
2'-SMe			2.15, s	2.07, s	2.22, s
5-OH			5.31, brs		
5'-OH			5.31, brs		
8-OH		5.68, s	5.87, s	5.36, brs	4.83, brs
8'-OH		5.41, brs	5.87, s	5.89, s	6.21, s

^a^ Measured in CDCl_3_, ^b^ Measured in DMSO-*d*_6_, ^c^ Measured in acetone-*d*_6_.

**Table 2:** ^13^C NMR (125 MHz) data of compounds **1**–**5** (δ in ppm).

Position	1 ^a^	2 ^b^	3 ^b^	4 ^b^	5 ^c^
1	165.9, C	163.7, C	168.5, C	169.0, C	166.9, C
2	74.0, C	74.7, C	72.4, C	71.1, C	72.4, C
3	31.6, CH_2_	38.4, CH_2_	35.0, CH_2_	31.2, CH_2_	35.5, CH_2_
4	48.1, CH	133.8, CH	43.3, CH	43.8, CH	45.5, CH
5	206.5, C	118.7, CH	68.8, CH	207.4, C	207.4, C
6	37.1, CH_2_	123.3, CH	133.3, CH	34.2, CH_2_	34.8, CH_2_
7	30.8, CH_2_	130.0, CH	129.9, CH	25.8, CH_2_	27.4, CH_2_
8	71.0, CH	73.8, CH	71.3, CH	64.7, CH	70.2, CH
9	69.9, CH	68.0, CH	67.8, CH	63.3, CH	67.2, CH
1'	169.6, C	169.0, C	168.5, C	165.2, C	170.4, C
2'	59.6, CH	58.8, CH	72.4, C	72.7, C	73.9, C
3'	27.1, CH_2_	29.2, CH_2_	35.0, CH_2_	31.0, CH_2_	32.5, CH_2_
4'	46.9, CH	46.0, CH	43.3, CH	47.0, CH	47.4, CH
5'	207.9, C	209.6, C	68.8, CH	206.8, C	196.4, C
6'	36.2, CH_2_	34.1, CH_2_	133.3, CH	36.7, CH_2_	129.1, CH
7'	26.7, CH_2_	26.6, CH_2_	129.9, CH	33.7, CH_2_	152.6, CH
8'	72.2, CH	64.0, CH	71.3, CH	71.1, CH	73.8, CH
9'	68.1, CH	65.0, CH	67.8, CH	69.0, CH	69.8, CH
2-SMe	14.5, CH_3_	13.2, CH_3_	14.3, CH_3_	14.1, CH_3_	14.9, CH_3_
2'-SMe			14.3, CH_3_	14.3, CH_3_	14.9, CH_3_

^a^ Measured in CDCl_3_; ^b^ Measured in DMSO-*d*_6_; ^c^ Measured in acetone-*d*_6_.

**Figure 3:**
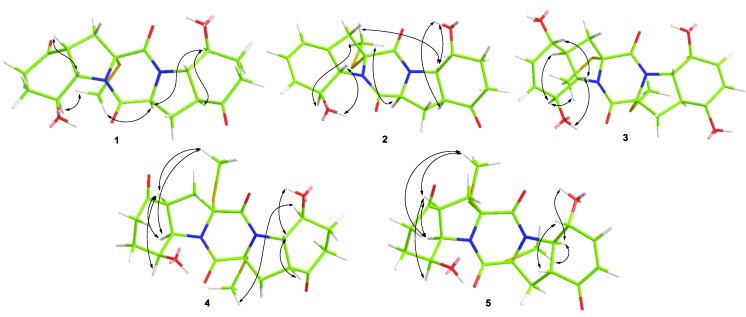
NOESY correlations of compounds **1**–**5**.

**Figure 4:**
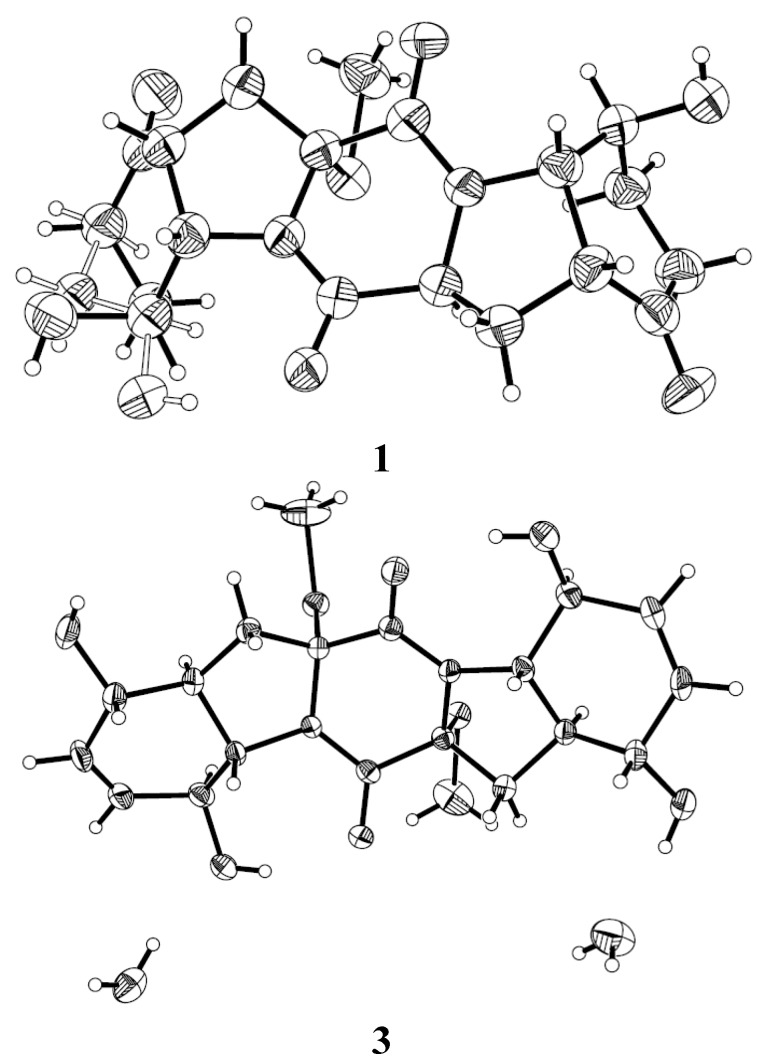
X-ray structure of compounds **1** and **3**.

The relative configuration of compound **1** was determined by analysis of its ^1^H-^1^H coupling constants and NOESY experiment. The coupling constants for H-4 and H-9 (*J*_H-4/H-9_ = 8.0 Hz) as well as for H-4' and H-9' (*J*_H-4__'__/H-9__'_ = 12.8 Hz), revealed the *cis*-relationship between H-4 and H-9 and *trans*-relationship of H-4' and H-9'. The NOE correlations from SCH_3_-2 to H-8, H-2', and H-8' as well as from H-8' to H-2' and H-4' indicated the same orientation of these groups (Figure 3). An X-ray crystallographic experiment (Figure 4) confirmed the structure and relative configuration of **1**. The Cu/Kα radiation allowed the assignment of the absolute configuration of all the stereogenic centers in **1** as 2*R*, 4*R*, 8*S*, 9*S*, 2'*S*, 4'*S*, 8'*S*, and 9'*S.* The Electronic Circular Dichroism (ECD) spectrum of **1** showed negative Cotton Effect (CE) at 254 nm and positive CE at 218 nm (Supplementary Information). The negative CE around 254 nm is indicative for the 2*R* configuration of TDKPs [12]. Based on the above data, the structure of **1** was determined and it was named as penicibrocazine A.

Compound **2** was obtained as yellowish solid. The HRESIMS experiment established its molecular formula C_19_H_2__2_N_2_O_5_S (10 degrees of unsaturation). The ^1^H and ^13^C NMR chemical shifts for the left portion of **2** were identical to those of **6** (phomazine B) [13], whereas the right portion closely matched to that of **1**. Detailed analysis of the ^1^H-^1^H COSY and HMBC correlations (Figure 2) confirmed the planar structure of **2**. The relative configuration of compound **2** was determined by analysis of the NOESY spectrum. The NOE correlation from 8-OH to H-9 indicated the opposite orientation of H-8 and H-9, whereas correlations from 8'-OH to H-4' and H-9' and from 2-SMe to H-8, H-2' and H-9' indicated the same face of these groups (Figure 3). The absolute configuration of **2** was determined by comparison of its CD spectrum with that of phomazine B [13]. Three chromophoric groups including the S-methyl group, skewed diene, and an allylic hydroxy group contributed to the long-wavelength band around 270 nm [12]. Compound **2** showed similar CEs to those of phomazine B, indicating that the absolute configuration of the left portion at C-2, C-8, and C-9 of **2** are the same as those of phomazine B. These data in conjunction with chemical shifts and NOESY correlations, as compared with those of **1** from C-1' to C-9', allowed the assignment of the absolute configuration of all the stereogenic centers in **2** as 2*R*, 8*S*, 9*S*, 2'*S*, 4'S, 8'*R*, and 9'*R.* The structure of **2** was thus elucidated and it was named as penicibrocazine B.

Penicibrocazine C (**3**) was obtained as colorless crystals and its molecular formula was determined as C_20_H_26_N_2_O_6_S_2_ (nine degrees of unsaturation) on the basis of HRESIMS data. Its ^1^H and ^13^C NMR spectroscopic data (Table 1 and Table 2) revealed the presence of one methyl, one methylene, six methines (with two olefinic and three oxygenated/nitrogenated) and two quaternary carbons (with one amide carbonyl). These data only accounted for half of the elemental composition, implying a symmetrical feature for **3**. The structure of **3** was deduced from exhaustive analyses of the ^1^H-^1^H COSY and HMBC spectra. In the ^1^H-^1^H COSY spectrum, correlations from H-3 to H-4, from H-4 to H-5 and H-9, from H-8 to H-9, and from H-6 to H-7 were observed, while in the HMBC spectrum, a full set of all possible ^2^*J* and ^3^*J* correlations, such as from H-3 to C-1, C-2, C-5, and C-9, from H-6 to C-4 and C-8, from H-7 to C-5, and from 5-OH to C-5, allowed for construction of the C-1 through C-9 fragment. The chemical shift of the methyl group (δ_H_/δ_C_ 2.15/14.3) and the HMBC cross-peak from the methyl protons to C-2 indicated that the methyl group was connected to the sulfur atom attached to C-2. These data in conjunction with symmetrical feature indicated the planar structure of **3** as shown in Figure 1.

The relative configuration of compound **3** was determined by analysis of its ^1^H-^1^H coupling constants and NOESY data. The large coupling constants between H-4 and H-9 (*J* = 11.9 Hz) revealed their *trans* relationship. NOE correlations from H-9 to OH-8 and H-5 in the NOESY spectrum indicated the cofacial orientation of these groups, while the observed correlations from H-8 to H-4 and 2-SMe placed these groups on the opposite face. An X-ray crystallographic experiment (Figure 4) confirmed the structure and relative configuration of **3** as depicted. The presence of sulfur in the molecule and the Flack parameter 0.02(9) allowed the assignment of the absolute configuration of all the stereogenic centers as 2*R*, 4*S*, 5*S*, 8*S*, and 9*S.*

Penicibrocazine D (**4**) was isolated as colorless solid. Its molecular formula was determined as C_20_H_26_N_2_O_6_S_2_ by HRESIMS data, having one SCH_2_ unit more than that of **1**. Detailed comparison of the NMR data of **4** with those of **1** suggested that compound **4** had the same basic 6-5-6-5-6 diketopiperazine skeleton as **1**. The NMR spectroscopic data (Table 1 and Table 2) differed from those of **1** mainly in the absence of the methine signal at δ_H_ 4.57 and δ_C_ 59.6 (C-2'). Instead, one *S*-methyl signal at δ_H_ 2.07 and δ_C_ 14.3 (2'-SMe) and one quaternary carbon signal at δ_C_ 72.7 (C-2') were observed in the NMR spectra of **4**. The substitution of a *S*-methyl group at C-2' of **4** was evidenced by the HMBC correlation from 2'-SMe to C-2'.

The relative configuration of **4** was assigned by *J*-coupling constants and NOESY spectrum. The NOE correlations from 2-SMe to H-4 and H-9 and from H-8 to H-4 as well as the coupling patterns for H-4 (*J* = 8.0 Hz) and H-9 (*J* = 8.0 Hz) indicated the cofacial relationship of these groups. The large coupling constant between H-8' and H-9' (*J* = 13.2 Hz) revealed their *trans* relationship. The *J* coupling constant between H-9' and H-4' (*J* = 8.8 Hz) and NOE correlations from H-9' to H-4' and OH-8' indicated the cofacial orientation of these protons, while the correlation from H-8' to 2'-SMe placed these protons on the opposite face. As the CD spectrum of **4** exhibited negative CEs at approximately 259 nm, the absolute configuration of the α/α' chiral centers were determined to be 2*R*/2'*R* [12]. Therefore, the absolute configuration of compound **4** was assigned as 2*R*, 4*S*, 8*S*, 9*R*, 2'*R*, 4'*R*, 8'*S*, and 9'*S*.

Penicibrocazine E (**5**) had the molecular formula C_20_H_24_N_2_O_6_S_2_ as determined by HRESIMS data, with two hydrogen atoms less than **4**. Detailed interpretation of the NMR data indicated that the ^1^H and ^13^C NMR chemical shifts for the left portion of **5** were nearly identical to those of **4**. For the right portion, the primary difference in the NMR spectroscopic data was that the signals for two alphatic methylenes at δ_C_ 36.7/δ_H_ 2.59 and 2.27 (CH_2_-6') and at δ_C_ 33.7/δ_H_ 1.58 and 2.27 (CH_2_-7') in **4** disappear in the NMR spectra of **5**. Instead, two olefinic methine signals at δ_C_ 129.1/δ_H_ 6.01 (CH-6') and δ_C_ 152.6/δ_H_ 6.86 (CH-7') were observed in the NMR spectra of **5**. These observations were supported by relevant ^1^H-^1^H COSY and HMBC correlations (Figure 2). Furthermore, the resonance for C-5' was more upfield (−10.4 ppm) in the ^13^C NMR spectrum of **5**, due to the incorporation of the conjugated double bond at C-6'.

The relative configuration of **5** was also determined by analysis of *J*-coupling constants and the NOESY spectrum. The coupling patterns for the relevant protons on the left portion of **5** were similar to their counterparts of **4**, indicating that the left motif of **5** possessed the same relative configuration as that of **4**. The large coupling constant between H-4' and H-9' (*J* = 13.0 Hz) revealed their *trans* relationship. NOE correlations from H-9' to 8'-OH and 2'-SMe placed these protons on the same face of the molecule, while the NOE correlation from H-8' to H-4' placed these protons on the opposite face. As might be expected, the CD spectrum of **5** exhibited negative CEs at approximately 260 nm, similar as that of **4**, indicating the absolute configuration of **5** to be 2*R*, 4*S*, 8*S*, 9*R*, 2'*R*, 4'*R*, 8'*R*, and 9'*R*.

### 2.2. Biological Activities of the Isolated Compounds

The isolated compounds **1**–**6** were examined for antimicrobial activity against several human-, aqua-, and plant-pathogenic microbes as well as cytotoxicity. In the antimicrobial screening, compounds **2**–**4** and **6** showed activity against *Staphylococcus aureus*, with MIC values of 32.0, 0.25, 8.0, and 0.25 μg/mL, respectively, which are comparable to that of the positive control, chloromycetin (MIC = 4.0 μg/mL). Compound **3** also showed activity against *Micrococcus luteus* with MIC value of 0.25 μg/mL, which is stronger than that of the positive control, chloromycetin (MIC = 2.0 μg/mL). Moreover, Compounds **2** and **4**–**6** exhibited activity against plant pathogen *Gaeumannomyces graminis* with MIC values of 0.25, 8.0, 0.25, and 64.0 μg/mL, respectively, while the positive control amphotericin B has MIC value of 16.0 μg/mL. These data indicated that the double bonds at C-6 and C-6' increased the activity against *S. aureus* (**3** *vs.* **1**). In addition, more *S*-methyl groups likely strengthened their activity against *G. graminis* (**4** *vs.* **1**), and keto groups at C-5/5' (**5** *vs.* **3**) also enhanced the activity. Compounds **1**–**6** were also evaluated for cytotoxicity against eight tumor cell lines (Du145, HeLa, HepG2, MCF-7, NCI-H460, SGC-7901, SW1990, and U251), but none of them displayed potent inhibitory activity (IC_50_ > 10 μM).

## 3. Experimental Section

### 3.1. General

Melting points were determined with an SGW X-4 micro-melting-point apparatus. Optical rotations were measured on an Optical Activity A-55 polarimeter. UV spectra were measured using a Lengguang Gold S54 spectrophotometer (Shanghai Lengguang Technology Co. Ltd., Shanghai, China). The ^1^H, ^13^C, and 2D NMR spectra were acquired using a Bruker Avance 500 spectrometers (Bruker Biospin Group, Karlsruhe, Germany). Mass spectra were measured on a VG Autospec 3000 mass spectrometer (VG Instruments, London, UK). Semi-preparative HPLC was performed using a Dionex UltiMate U3000 system (Dionex Corporation, Sunnyvale, CA, USA) with an Agilent Prep RP-18 column (21.2 × 250 mm, 10 μm) under UV detection. Column chromatography (CC) was performed with silica gel (200–300 mesh, Qingdao Haiyang Chemical Factory, Qingdao, China), Lobar LiChroprep RP-18 (40–60 μm, Merck, Darmstadt, Germany), and Sephadex LH-20 (18–110 μm, Merck).

### 3.2. Fungal Material

The fungus *Penicillium brocae* MA-231 was isolated from the fresh tissue of the marine mangrove plant *Avicennia marina*. The fungus was identified by sequence analysis of the ITS region of its rDNA as described previously [10,14]. The resulting sequence data obtained from the fungal strain have been deposited in GenBank (with accession no KM191342). A BLAST search result indicated that the sequence was most similar (99%) to the sequence of *Penicillium brocae* (compared to AF484393). The strain is preserved at the China General Microbiological Culture Collection Center.

### 3.3. Fermentation

The fermentation was carried out statically in liquid potato-dextrose broth medium (PDB) (1000 mL seawater, 20 g glucose, 5 g peptone, 3 g yeast extract, and 200 g potato juice, pH 6.5–7.0, liquid medium/flask = 300 mL) in 1 L Erlenmeyer flasks for 30 days at room temperature.

### 3.4. Extraction and Isolation

The fermented whole broth (60 flasks) was filtered through cheesecloth to separate the culture broth and mycelia, which were exhaustively extracted with EtOAc and acetone, respectively. Since the chemical profiles of the two extracts were almost identical, they were combined and concentrated to afford the crude extract (20.0 g), which was subjected to silica gel vacuum liquid chromatography (VLC) eluting with mixed solvents of increasing polarity (petroleum ether to MeOH) to yield nine fractions (Fr.1 to Fr.9). Fraction 3 (1.5 g) was further purified by reverse-phase column chromatography (CC) over Lobar LiChroprep RP-18 with a MeOH-H_2_O gradient (from 20:80 to 100:0) to afford four subfractions Fr.3.1–Fr.3.4. Fr.3.2 was further purified by CC on Sephadex LH-20 (MeOH) and then by prep. TLC (plate: 20 × 20 cm, developing solvents: CHCl_3_/MeOH, 30:1) to obtain compounds **2** (7.9 mg) and **6** (11.9 mg). Fraction 5 (3.5 g) was further purified by reverse-phase column chromatography (CC) over Lobar LiChroprep RP–18 with a MeOH-H_2_O gradient (from 20:80 to 100:0) to afford six subfractions Fr.5.1–Fr.5.6. Fr.5.2 was further purified by CC on Sephadex LH-20 (MeOH), and on silica gel (eluted with CHCl_3_-MeOH, 100:1 to 5:1) to afford **1** (19.0 mg). Fr.5.3 was purified by CC on silica gel eluted with a CHCl_3_-MeOH gradient (from 100:1 to 10:1) and further purified by semi-preparative HPLC (MeOH-H_2_O 5:5, 16 mL/min) to afford **4** (*t*_R_ = 12.1 min, 3.6 mg). Fr.5.4 was subjected to CC on silica gel eluted with CHCl_3_-MeOH (100:1 to 5:1) and Sephadex LH-20 (MeOH) to obtain **5** (3.0 mg). Further purification of Fr. 6 (2.5 g) by CC over Lobar LiChroprep RP-18 with a MeOH-H_2_O gradient (from 20:80 to 100:0) afforded six subfractions. Fr. 6**-**1 was further purified by CC on Sephadex LH-20 (MeOH) to afford compound **3** (17.7 mg).

Penicibrocazine A (**1**): colorless crystals; mp 208–210 °C; ${\text{[α]}}_{20}^{\text{D}}$: −147.8 (*c* 0.46, MeOH); UV (MeOH) λ_max_ (log ε) 203 (4.60) nm; CD λ_max_ (Δε) 218 (+3.68), 254 (−9.01) nm; ^1^H and ^13^C NMR data, see Table 1 and Table 2; ESIMS *m/z* 409 [M + H]^+^; HRESIMS *m/z* 409.1428 [M + H]^+^ (calcd for C_19_H_2__5_N_2_O_6_S, 409.1428, Δ 0.0 ppm).

Penicibrocazine B (**2**): yellowish solid; ${\text{[α]}}_{20}^{\text{D}}$: −64.0 (*c* 0.10, MeOH); UV (MeOH) λ_max_ (log ε) 205 (4.12), 266 (3.65) nm; CD λ_max_ (Δε) 222 (−15.42), 273 (+5.26) nm; ^1^H and ^13^C NMR data, see Table 1 and Table 2; ESIMS *m/z* 391 [M + H]^+^; HRESIMS *m/z* 391.1329 [M + H]^+^ (calcd for C_19_H_2__3_N_2_O_2_S, 391.1322, Δ 0.7 ppm).

Penicibrocazine C (**3**): colorless crystals; mp 150–152 °C; ${\text{[α]}}_{20}^{\text{D}}$: −133.3 (*c* 0.28, MeOH); UV (MeOH) λ_max_ (log ε) 201 (4.64) nm; CD λ_max_ (Δε) 201 (+9.58), 216 (−8.38), 234 (+0.29), 259 (−8.37) nm; ^1^H and ^13^C NMR data, see Table 1 and Table 2; ESIMS *m/z* 455 [M + H]^+^; HRESIMS *m/z* 455.1307 [M + H]^+^ (calcd for C_2__0_H_2__7_N_2_O_6_S_2_, 455.1305, Δ 0.2 ppm).

Penicibrocazine D (**4**): yellowish solid; ${\text{[α]}}_{20}^{\text{D}}$: −80.0 (*c* 0.14, MeOH); UV (MeOH) λ_max_ (log ε) 206 (4.13) nm; CD λ_max_ (Δε) 200 (−16.31), 233 (+22.79), 259 (−17.89) nm; ^1^H and ^13^C NMR data, see Table 1 and Table 2; ESIMS *m/z* 455 [M + H]^+^; HRESIMS *m/z* 455.1296 [M + H]^+^ (calcd for C_2__0_H_2__7_N_2_O_6_S_2_, 455.1305, Δ 0.9 ppm).

Penicibrocazine E (**5**): yellowish solid; ${\text{[α]}}_{20}^{\text{D}}$: −176.0 (*c* 0.10, MeOH); UV (MeOH) λ_max_ (log ε) 206 (4.24) nm; CD λ_max_ (Δε) 212 (−22.48), 236 (+16.21), 261 (−15.03) nm; ^1^H and ^13^C NMR data, see Table 1 and Table 2; ESIMS *m/z* 453 [M + H]^+^; HRESIMS *m/z* 453.1149 [M + H]^+^ (calcd for C_2__0_H_2__5_N_2_O_6_S_2_, 453.1145, Δ 0.4 ppm).

### 3.5. X-Ray Crystallographic Analysis of Compounds **1** and **3** [15]

All crystallographic data were collected on a Bruker Smart-1000 CCD diffractometer equipped with a graphite-monochromatic Cu K*α* radiation (λ = 1.54178 Å) for **1** and Mo Kα radiation (λ = 0.71073 Å) for **3** at 293(2) K. The data were corrected for absorption by using the program SADABS [16]. The structures were solved by direct methods with the SHELXTL software package [17]. All non-hydrogen atoms were refined anisotropically. The H atoms were located by geometrical calculations, and their positions and thermal parameters were fixed during the structure refinement. The structure was refined by full-matrix least-squares techniques [18].

*Crystal data for compound* ***1***: C_1__9_H_2__3_N_2_O_6_S, F.W. (Formula Weight) = 407.45, orthorhombic space group, P2(1)2(1)2(1), unit cell dimensions *a* = 6.4278(12) Å, *b* = 13.001(3) Å, *c* = 23.565(4) Å, *V* = 1969.3(6) Å^3^, α = β = γ = 90°, *Z* = 4, d_calcd_ = 1.374 mg/m^3^, crystal dimensions 0.11 × 0.08 × 0.05 mm, μ = 1.800 mm^−^^1^, *F*(000) = 860. The 3943 measurements yielded 2894 independent reflections after equivalent data were averaged, and Lorentz and polarization corrections were applied. The final refinement gave *R*_1_ = 0.1273 and w*R*_2_ = 0.3072 [*I* > 2σ(*I*)]. The Flack parameter was 0.02(12) in the final refinement for all 3943 reflections with 2894 Friedel pairs.

*Crystal data for compound* ***3***: C_20_H_30_N_2_O_8_S_2_, F.W. = 490.58, orthorhombic space group, P2(1)2(1)2(1), unit cell dimensions *a* = 8.3513(7) Å, *b* = 11.8611(12) Å, *c* = 24.024(3) Å, *V* = 2379.7(4) Å^3^, α = β = γ = 90°, *Z* = 4, d_calcd_ = 1.369 mg/m^3^, crystal dimensions 0.38 × 0.32 × 0.10 mm, *μ* = 0.271 mm^−^^1^, *F*(000) = 1040. The 11941 measurements yielded 4180 independent reflections after equivalent data were averaged, and Lorentz and polarization corrections were applied. The final refinement gave *R*_1_ = 0.0445 and w*R*_2_ = 0.0893 [*I* > 2σ(*I*)].

### 3.6. Cytotoxicity Assay

The cytotoxic activity of the isolated compounds against nine tumor cell lines including Du145 (human carcinoma of prostate cell line), HeLa (human cervix carcinoma cell line), HepG2 (human liver hepatocellular cells), MCF-7 (human breast carcinoma cell line), NCI-H460 (human large cell lung carcinoma cell line), SGC-7901 (human gastric carcinoma cell line), SW1990 (human pancreatic cancer cell line), SW480 (human colon carcinoma cancer), and U251 (human glioma cells) were determined according to previously reported methods [19].

### 3.7. Antimicrobial Assay

Antimicrobial assay against human- and aqua-pathogenic microbes *Aeromonas hydrophilia*, *Escherichia coli*, *Micrococcus luteus*, *Staphylococcus aureus*, *Vibrio arveyi*, *V. parahaemolyticus* and plant pathogenic fungi *Alternaria brassicae*, *Colletotrichum gloeosporioides*, *Fusarium graminearum*, and *Gaeumannomyces graminis* was carried out using the well diffusion method [20]. Chloromycetin was used as a positive control for the bacteria, while amphotericin B was used as a positive control for the fungi.

## 4. Conclusions

Five new thiodiketopiperazine derivatives (**1**–**5**), along with one known analog (**6**), were isolated from the marine mangrove-derived endophytic fungus *P*. *brocae* MA-231. The structures and relative configurations were determined on the interpretation of the NMR data and the absolute configurations of all compounds were determined by CD comparison, while the structures of compounds **1** and **3** were confirmed by single-crystal X-ray diffraction analysis. Compounds **2**–**6** exhibited activity against some of the tested microbial strains.

## Supplementary Files

Supplementary File 1
