# Supplementary material for: Penicibrocazines A–E, Five New Sulfide Diketopiperazines from the Marine-Derived Endophytic Fungus Penicillium brocae
Source: Mar Drugs. 2015 Jan 7;13(1):276–87. doi: 10.3390/md13010276 (PMC4306937; doi:10.3390/md13010276)
Supplement: Supplementary File 1 [file marinedrugs-13-00276-s001.pdf]

Supplementary Information

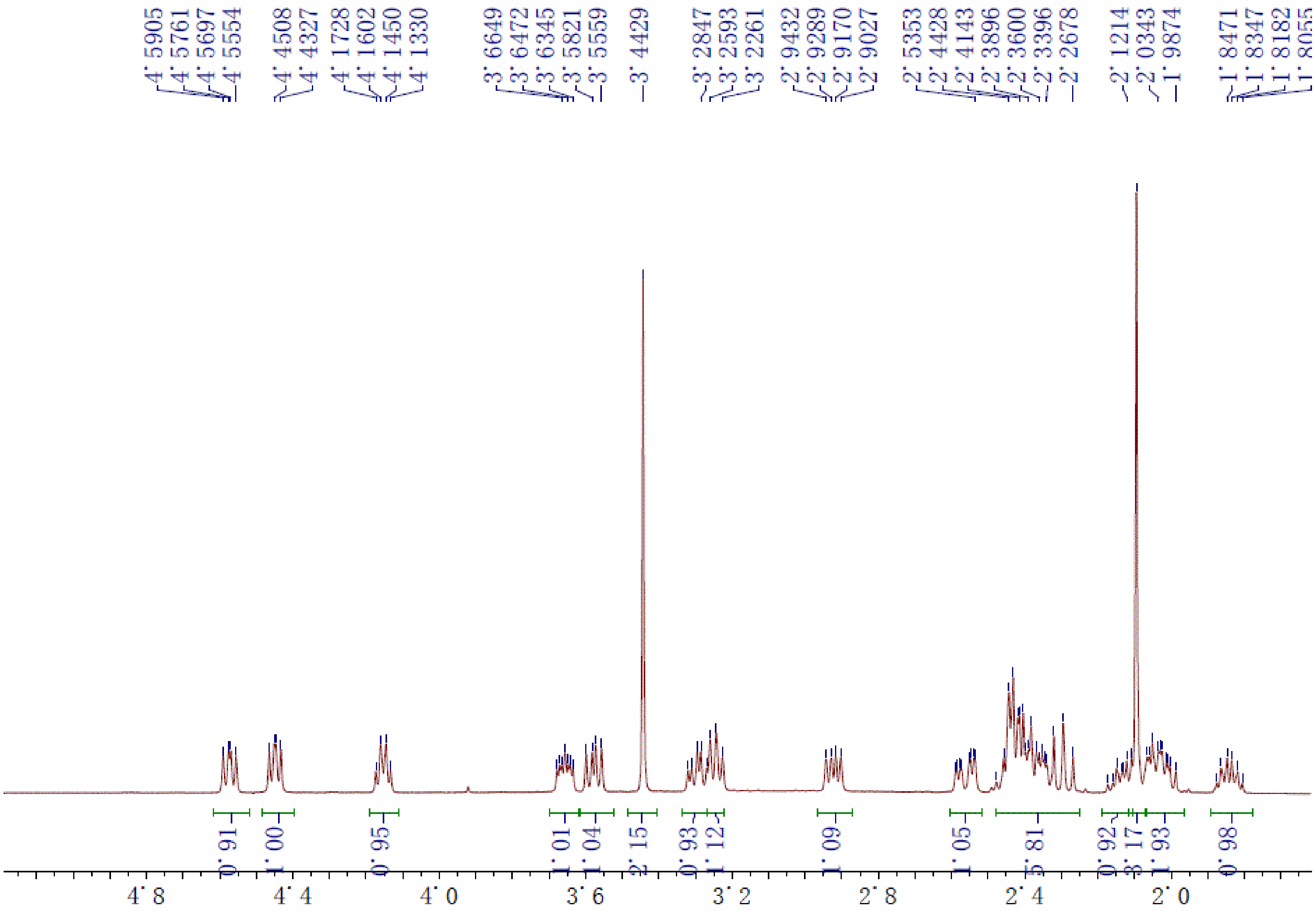

Figure S1. ^{1}H NMR (500 MHz, $CDCl_3$) spectrum of compound **1**.

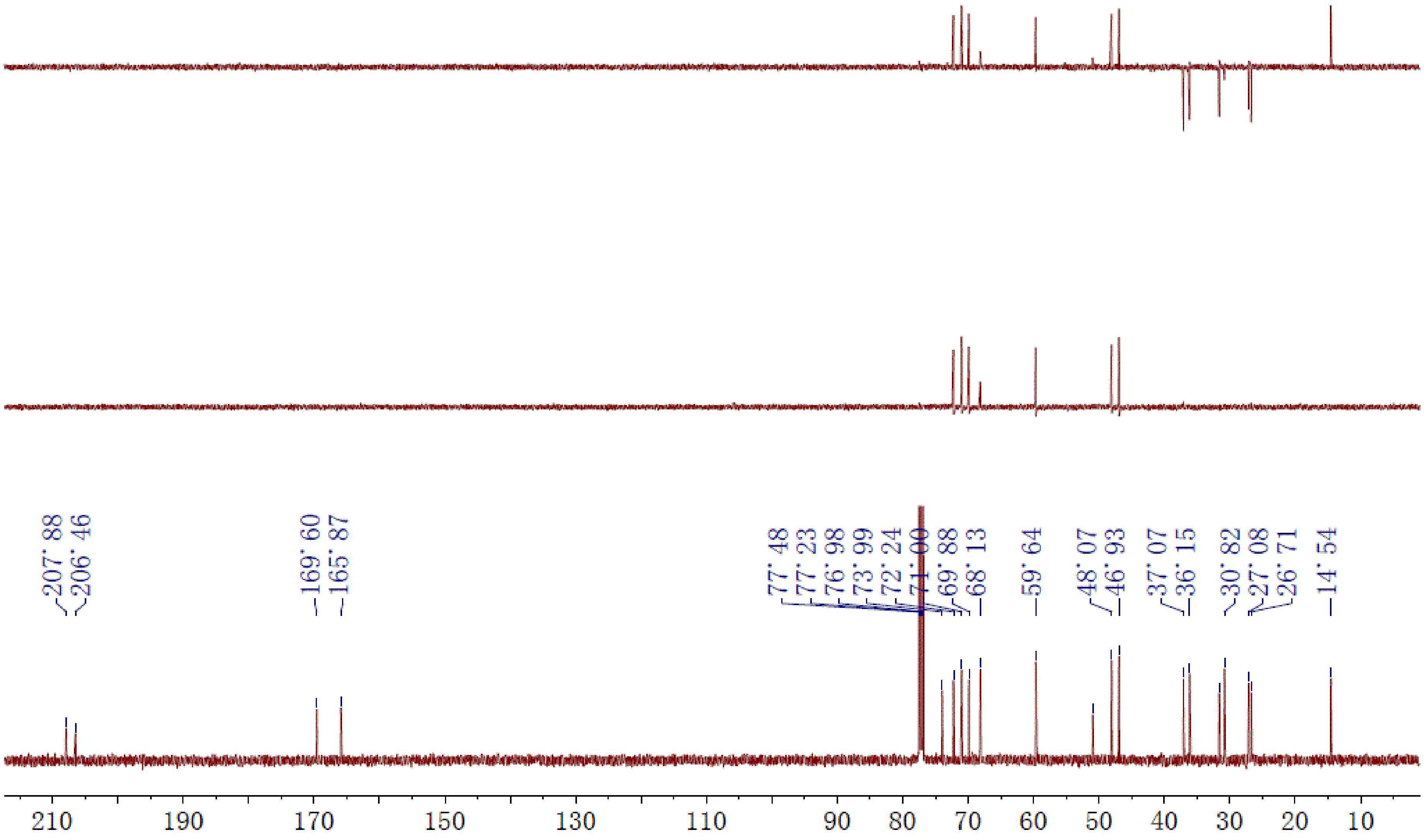

Figure S2. ^{13}C NMR (125 MHz, $CDCl_3$) and DEPT spectra of compound **1**.

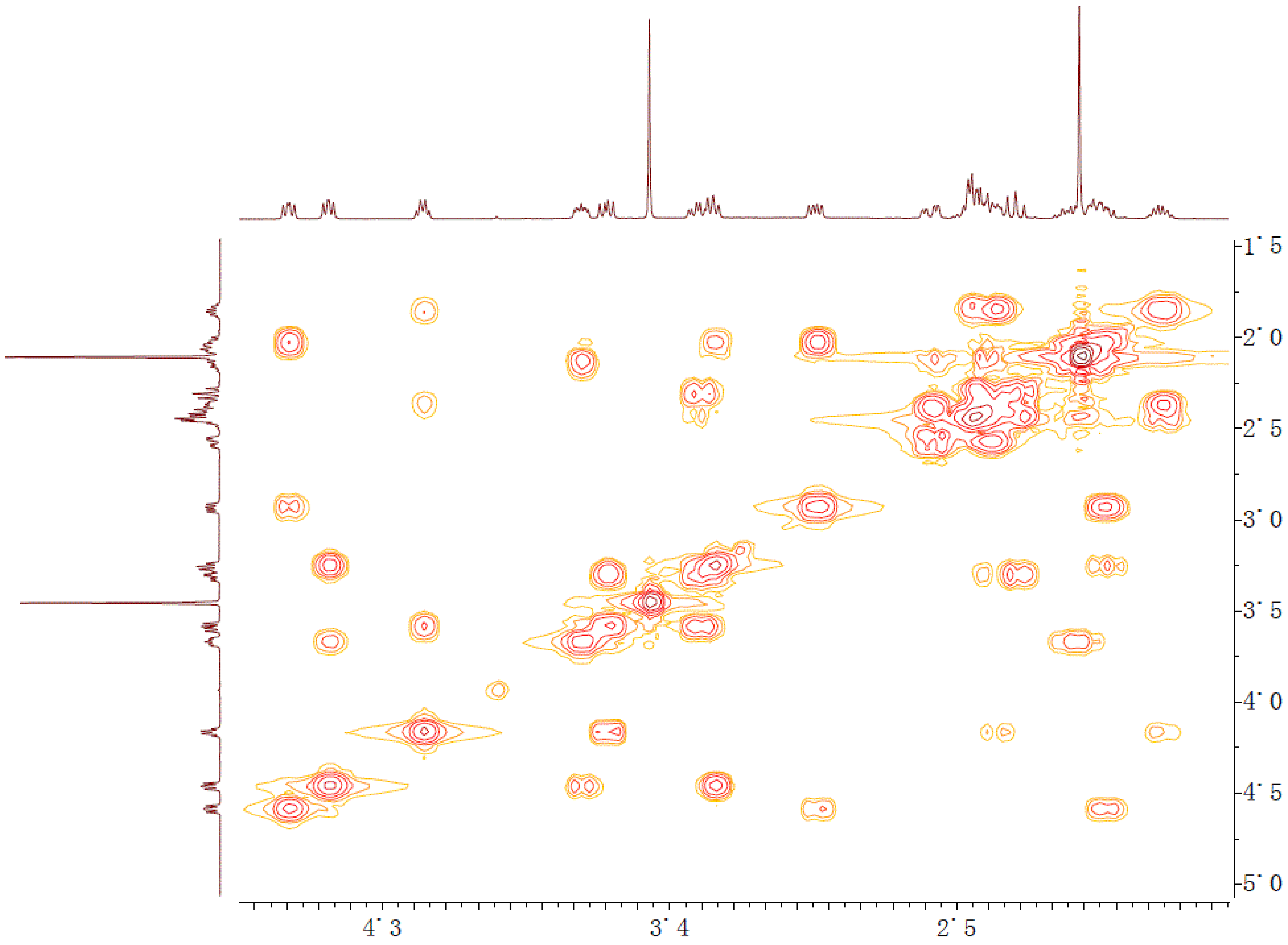

Figure S3. COSY spectrum of compound **1**.

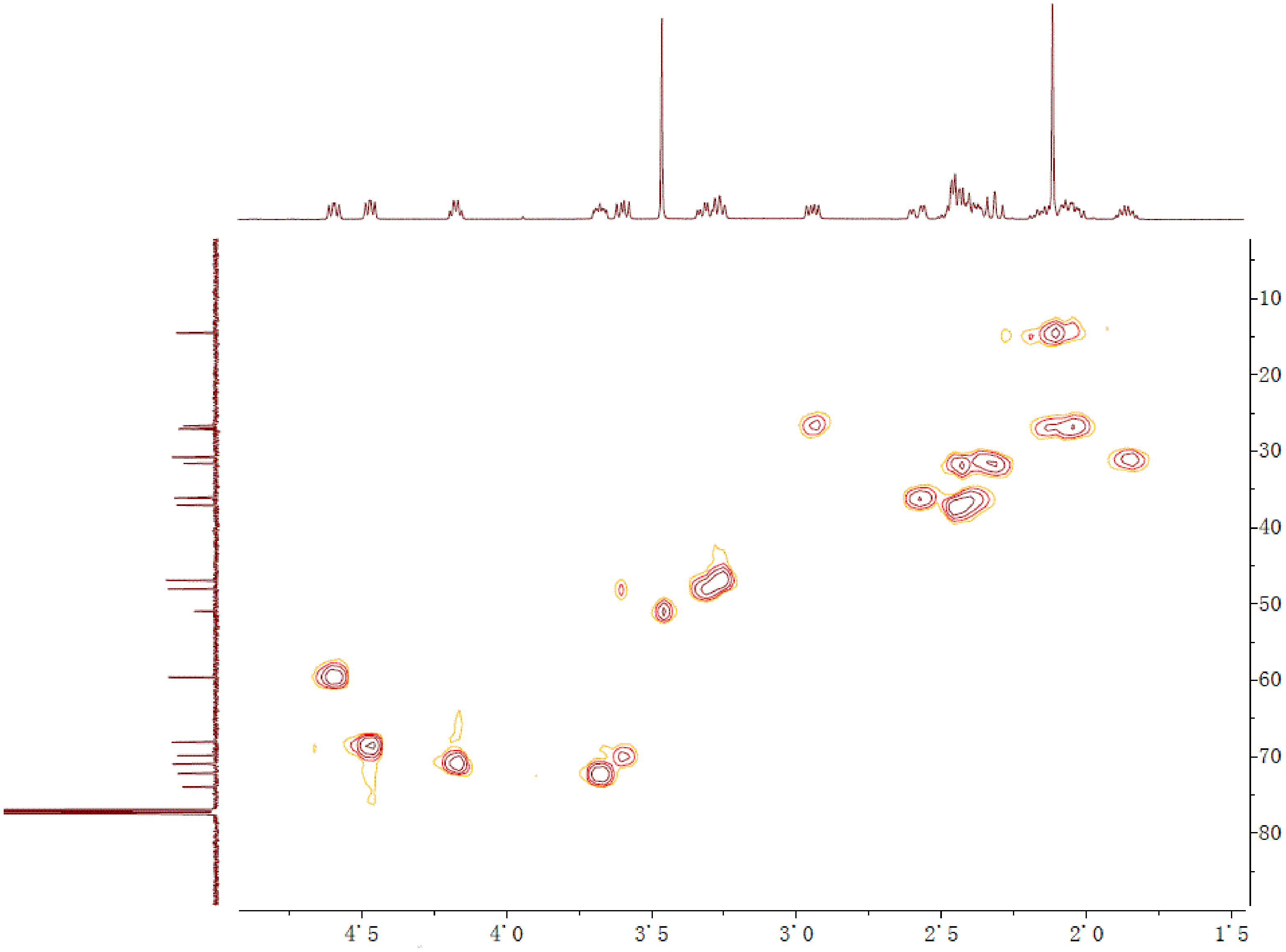

Figure S4. HSQC spectrum of compound **1**.

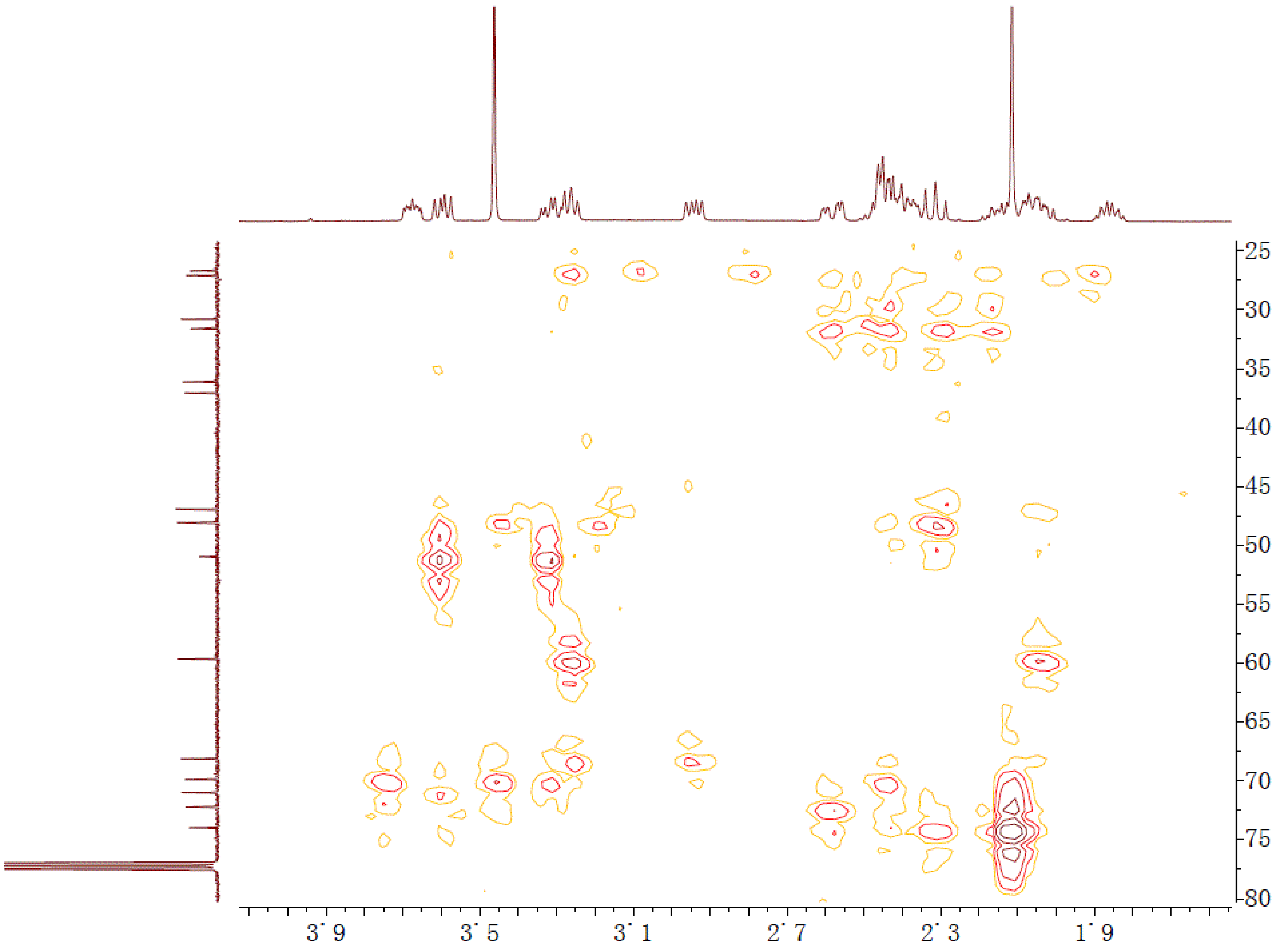

Figure S5. HMBC spectrum of compound **1**.

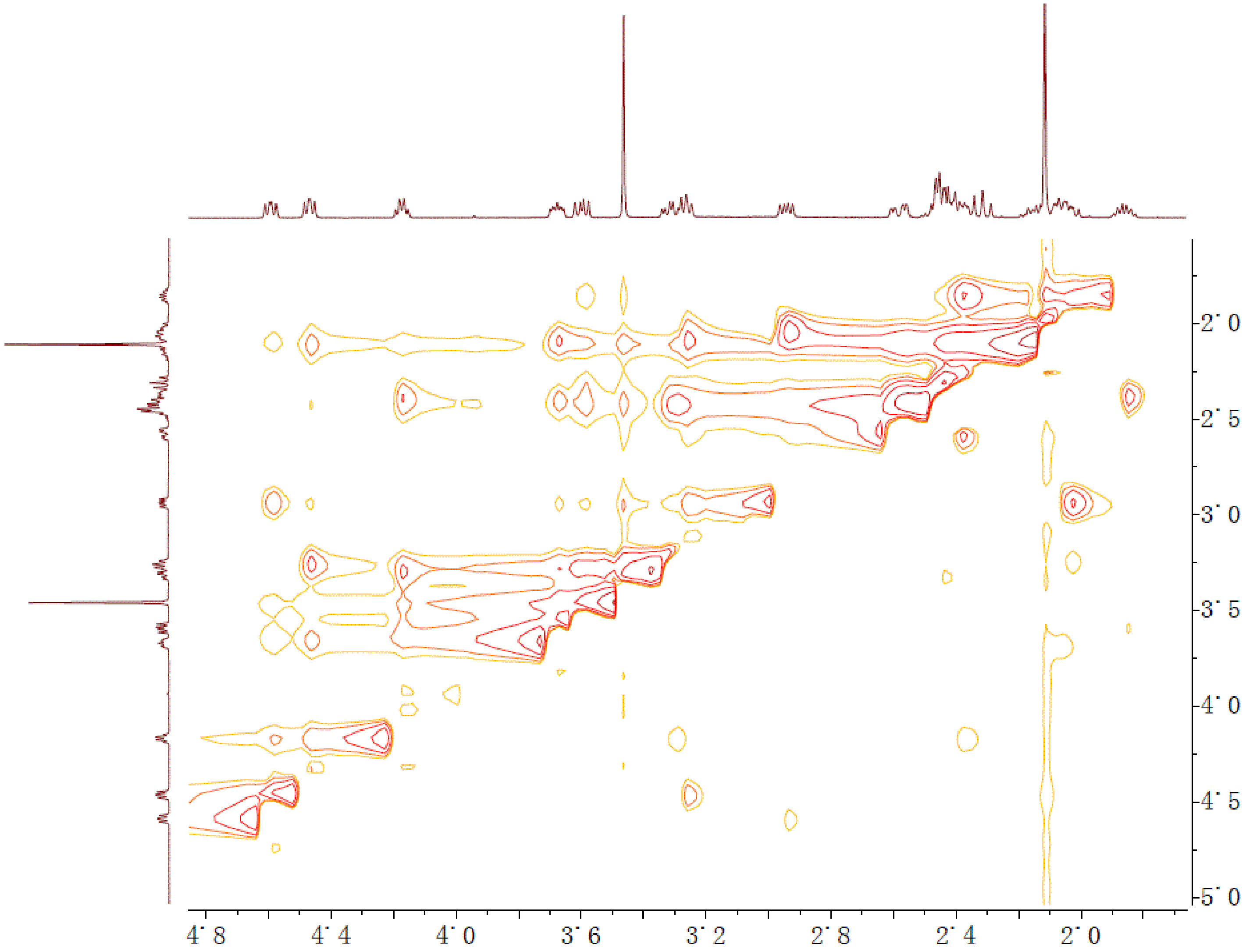

Figure S6. NOESY spectrum of compound **1**.

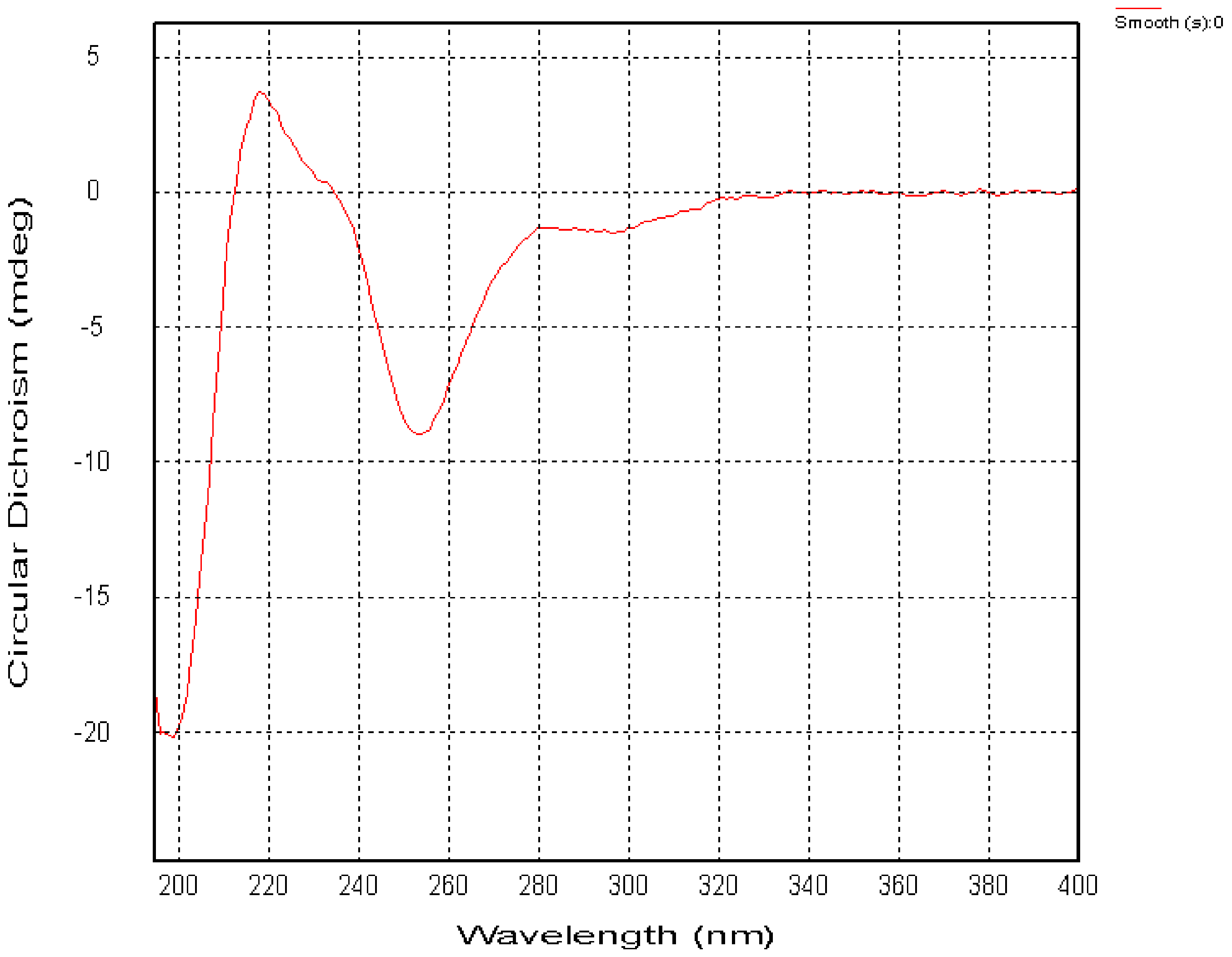

Figure S7. CD spectrum of compound **1**.

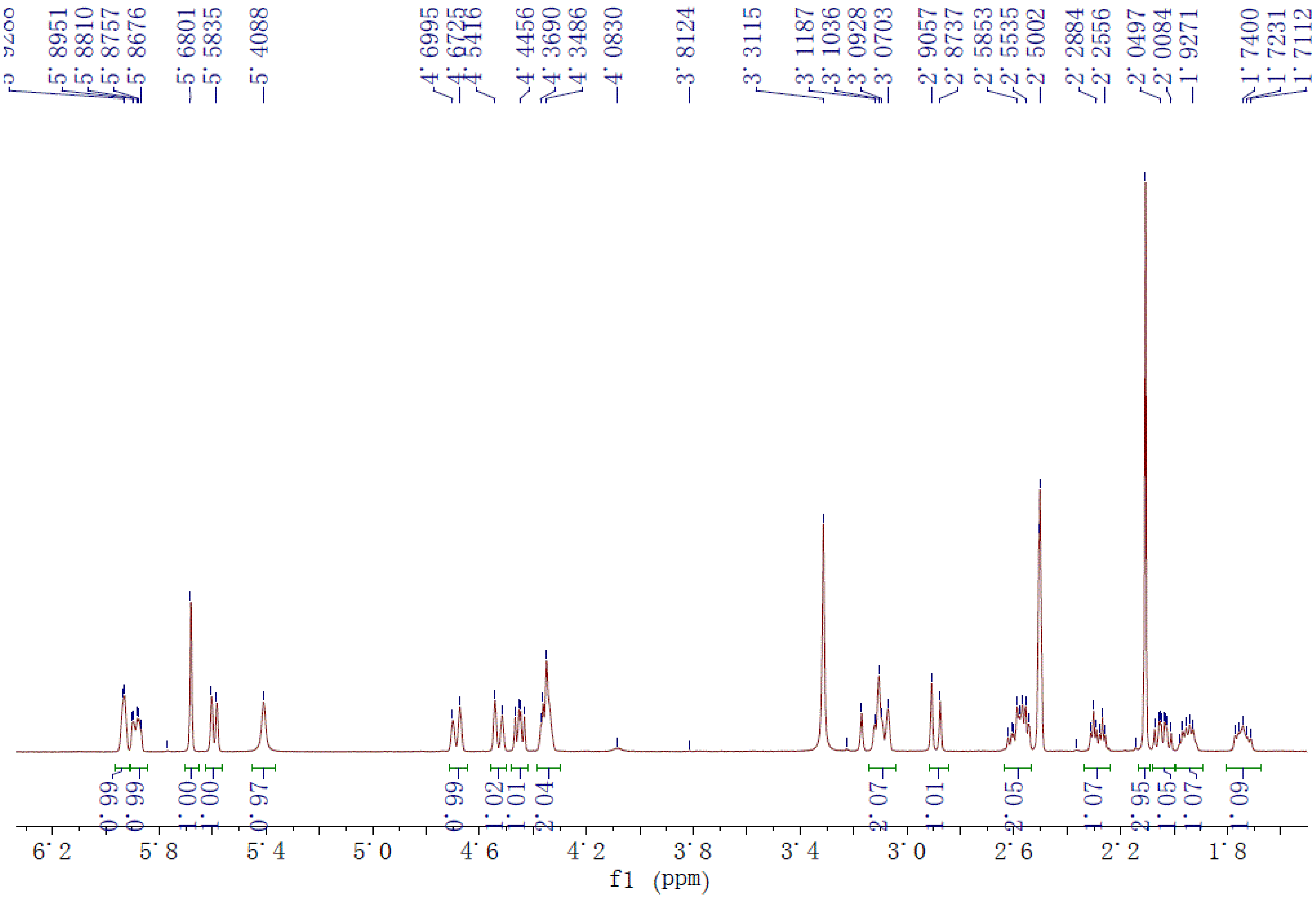

Figure S8. ^1H NMR (500 MHz, DMSO-d_6) spectrum of compound **2**.

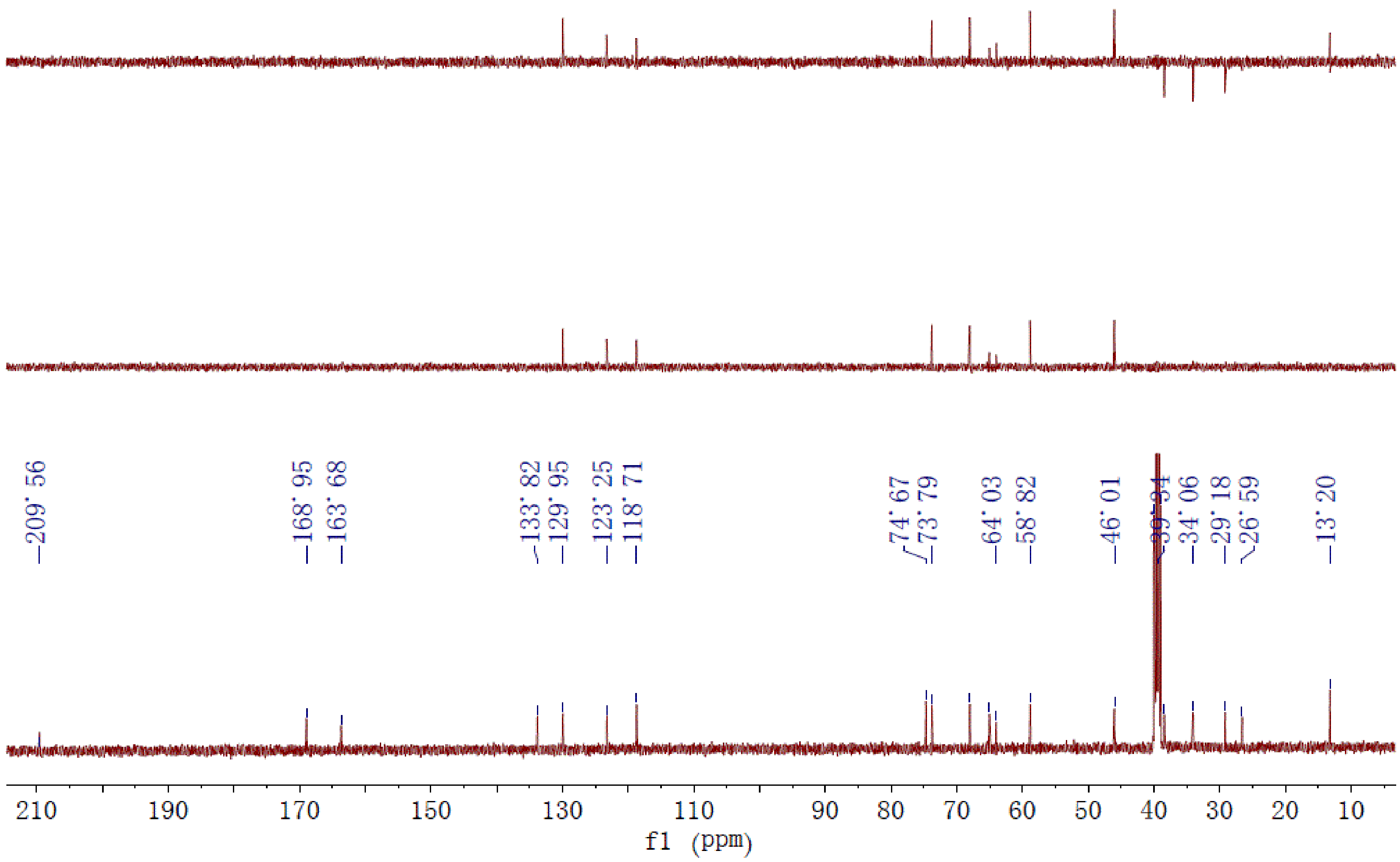

Figure S9. ^{13}C NMR (125 MHz, DMSO-d_6) and DEPT spectra of compound **2**.

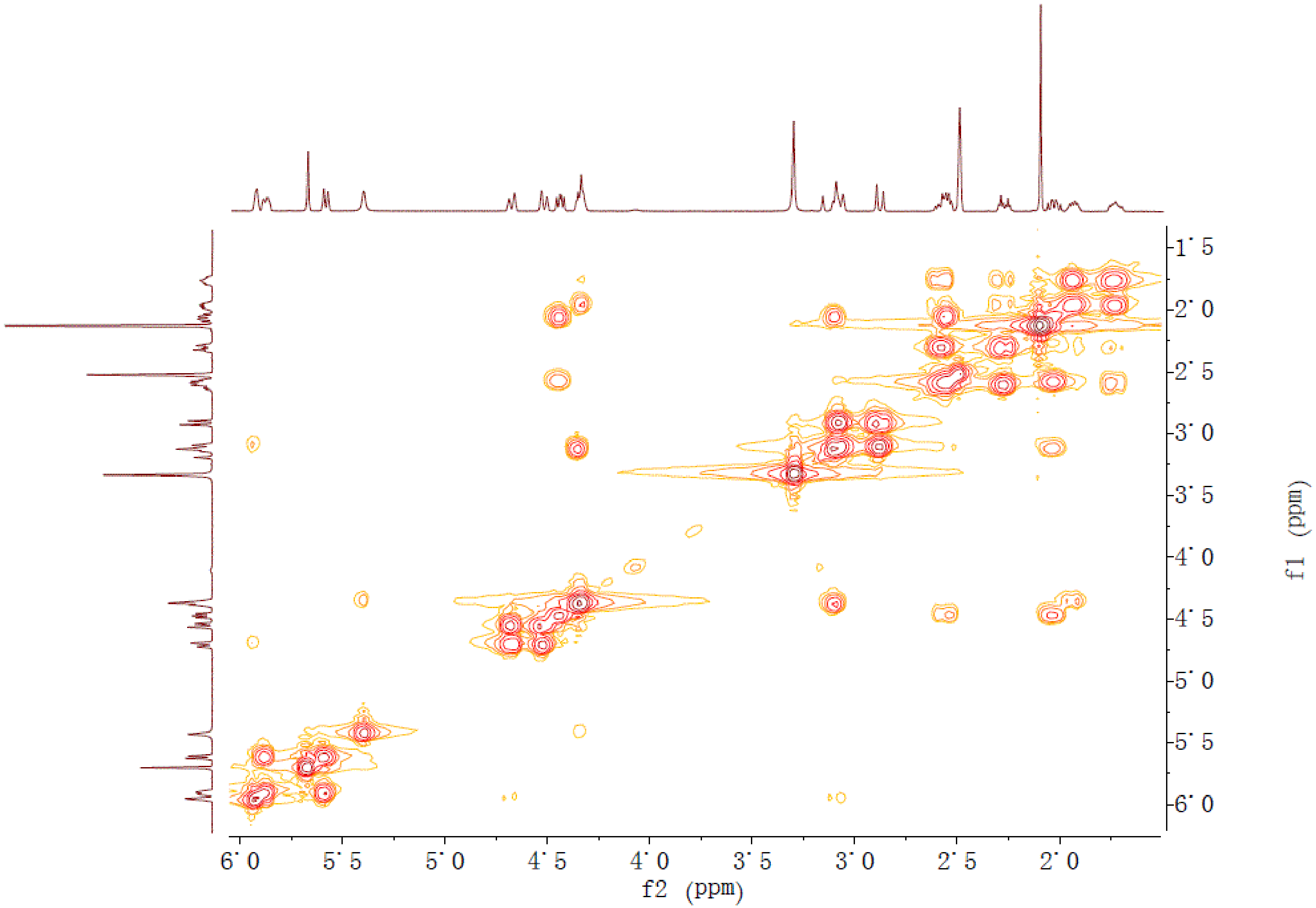

Figure S10. COSY spectrum of compound **2**.

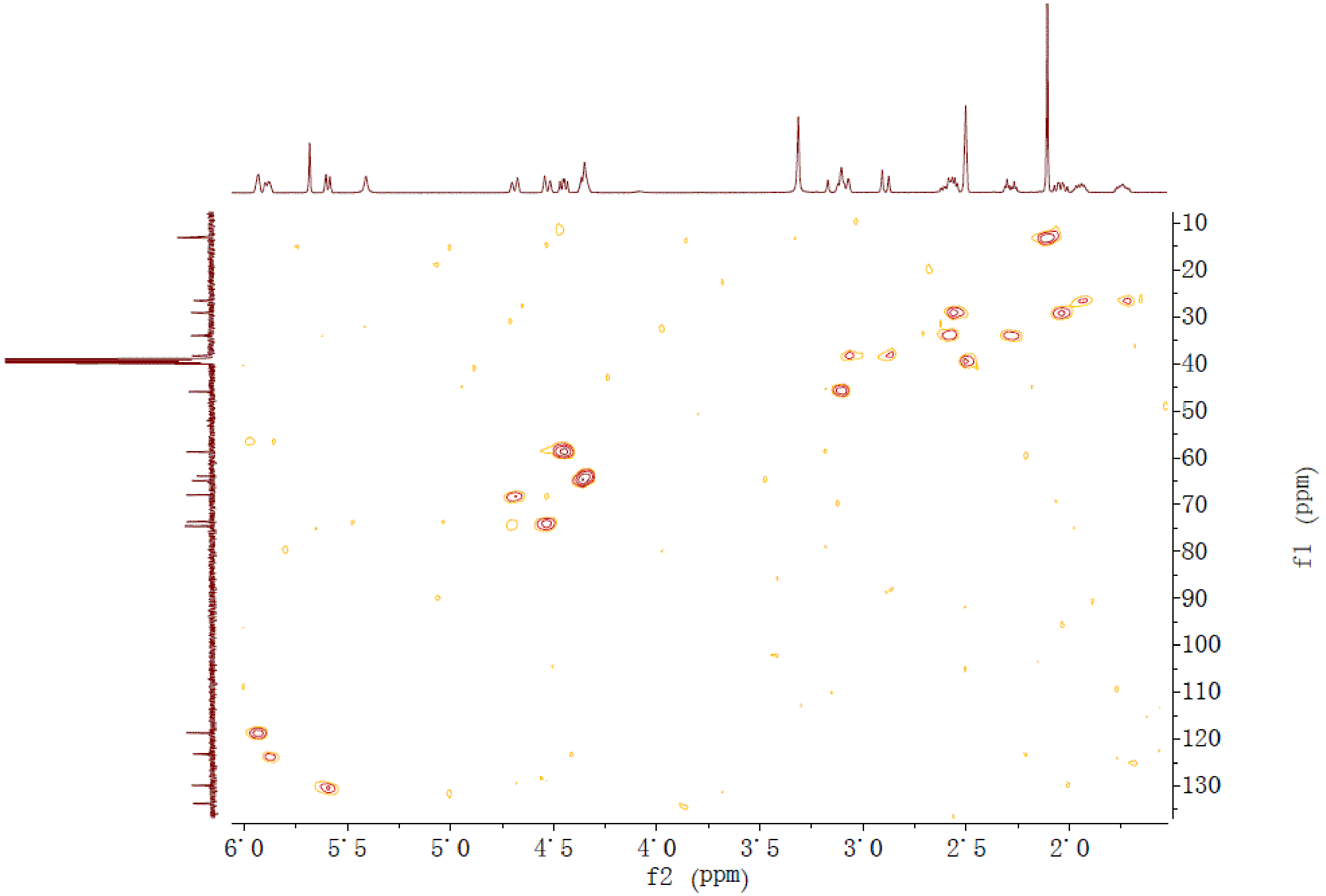

Figure S11. HSQC spectrum of compound **2**.

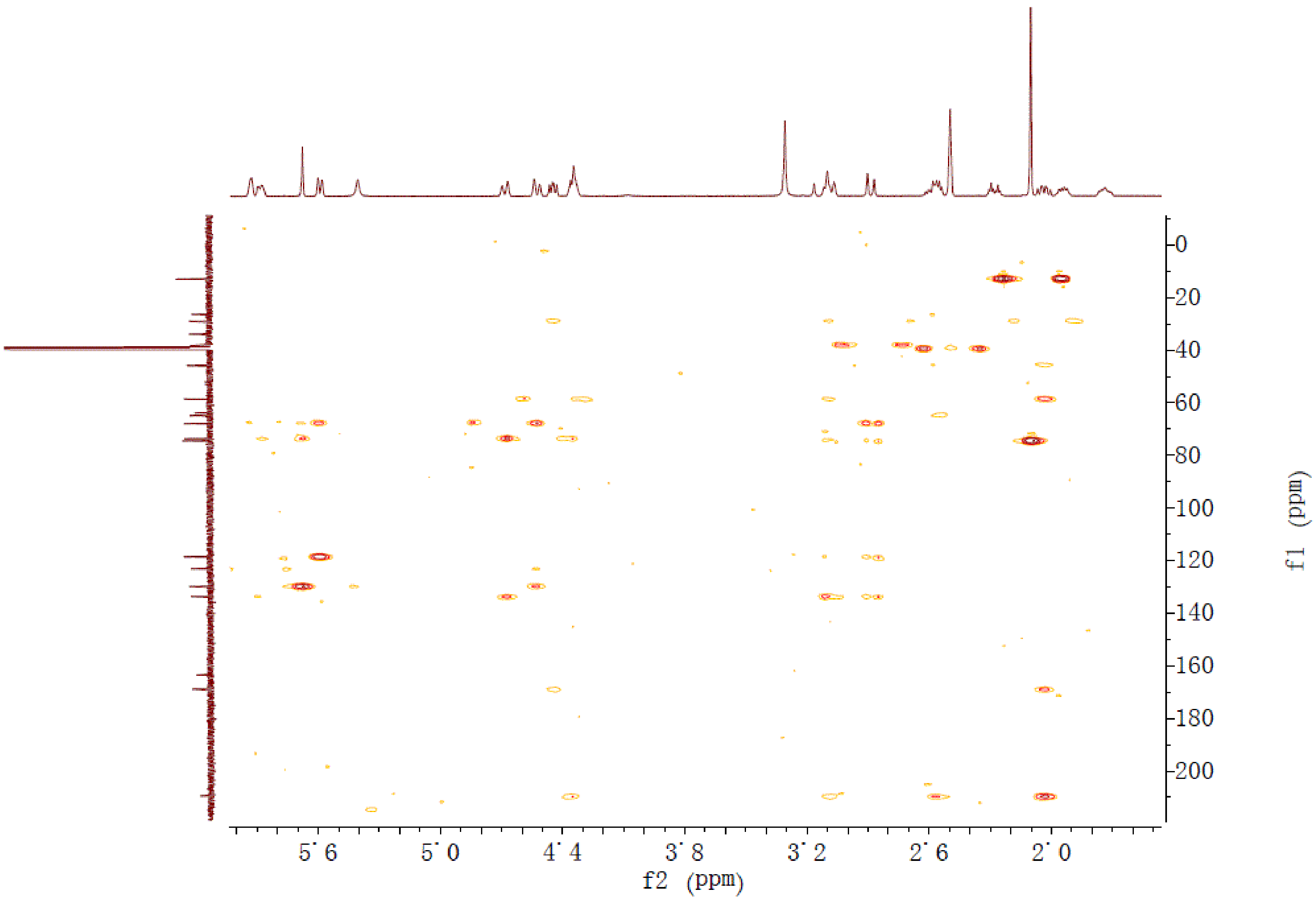

Figure S12. HMBC spectrum of compound **2**.

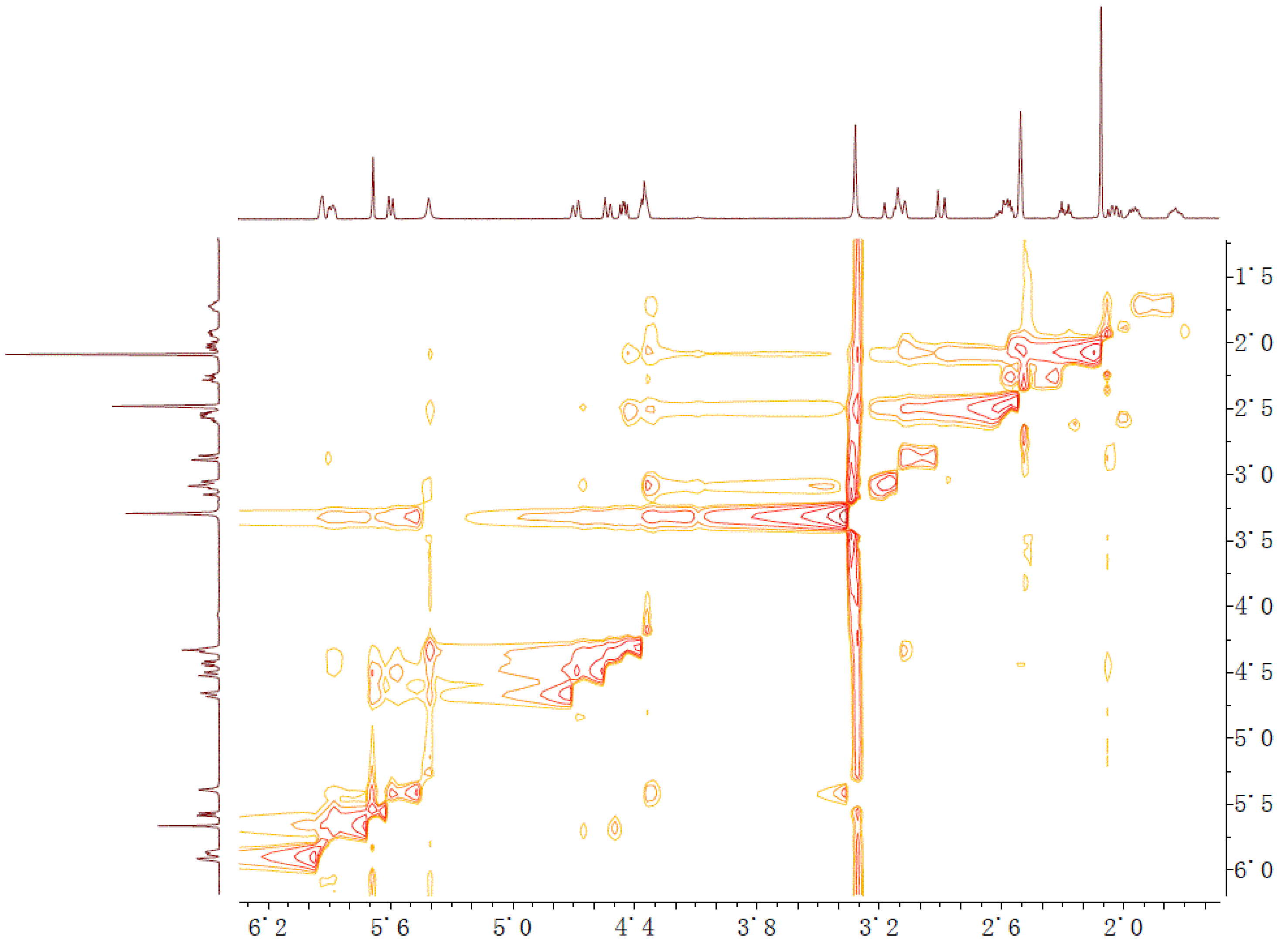

Figure S13. NOESY spectrum of compound **2**.

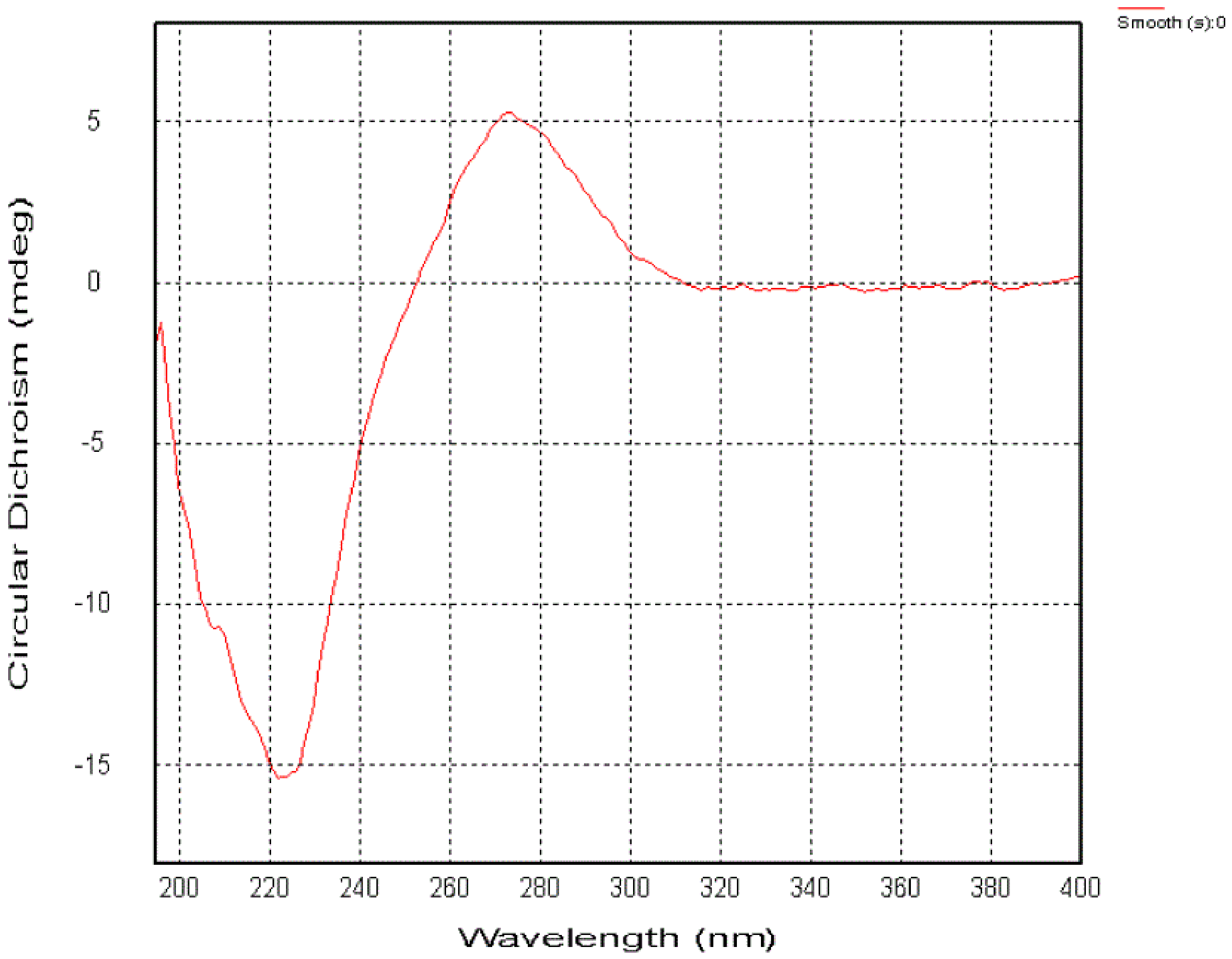

Figure S14. CD spectrum of compound **2**.

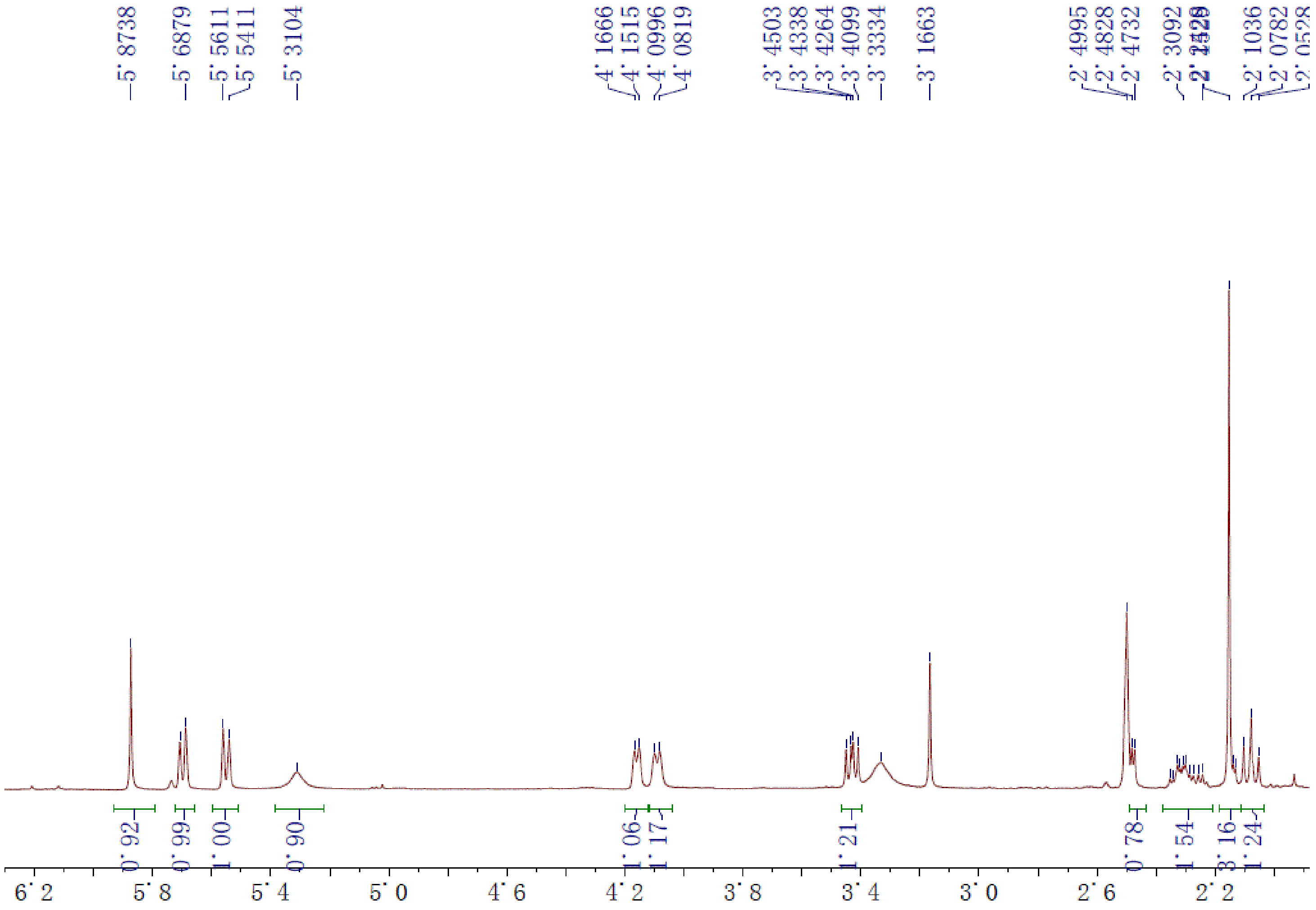

Figure S15. ^{1}H NMR (500 MHz, DMSO-d_6) spectrum of compound **3**.

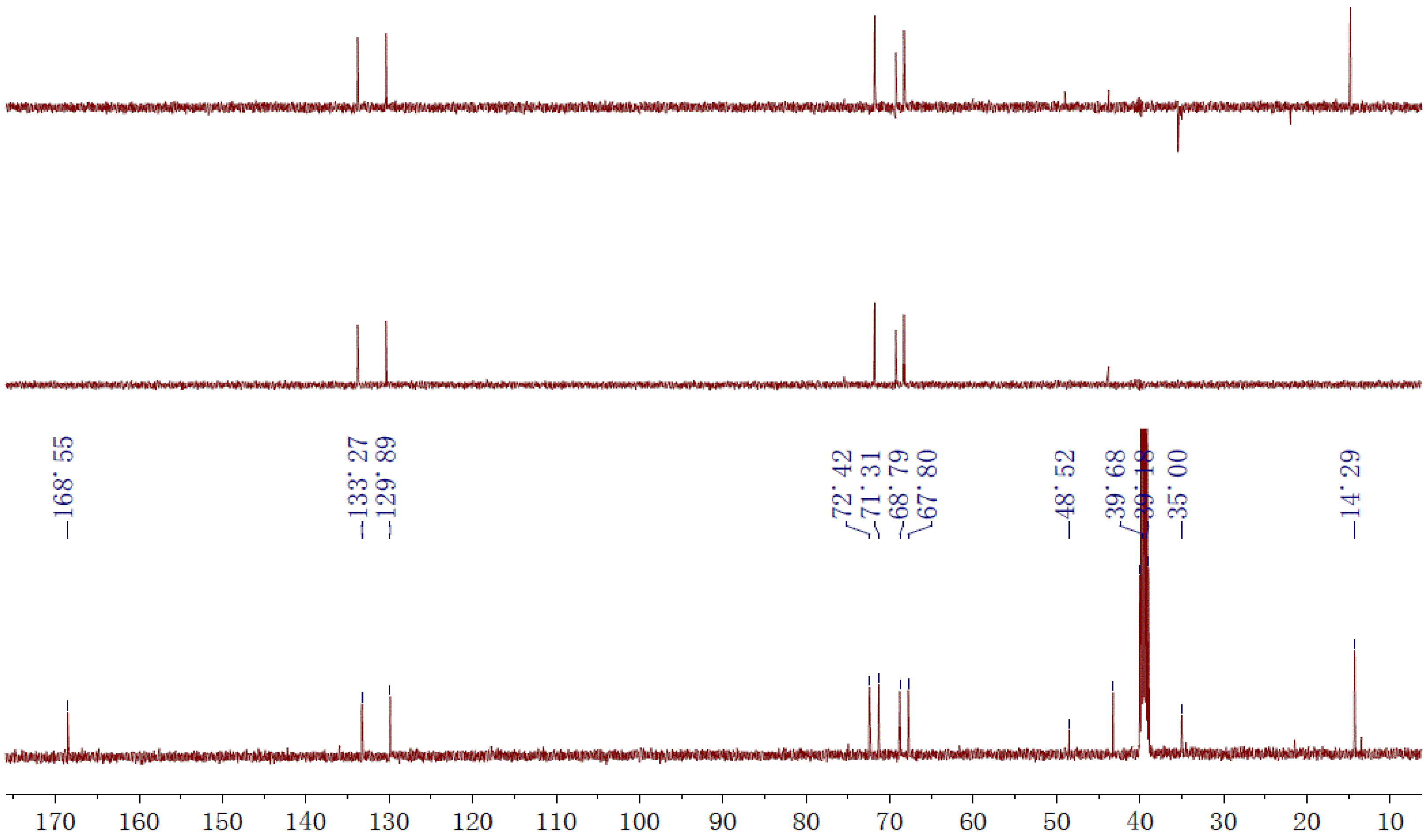

Figure S16. ^{13}C NMR (125 MHz, DMSO-d_6) and DEPT spectra of compound **3.**

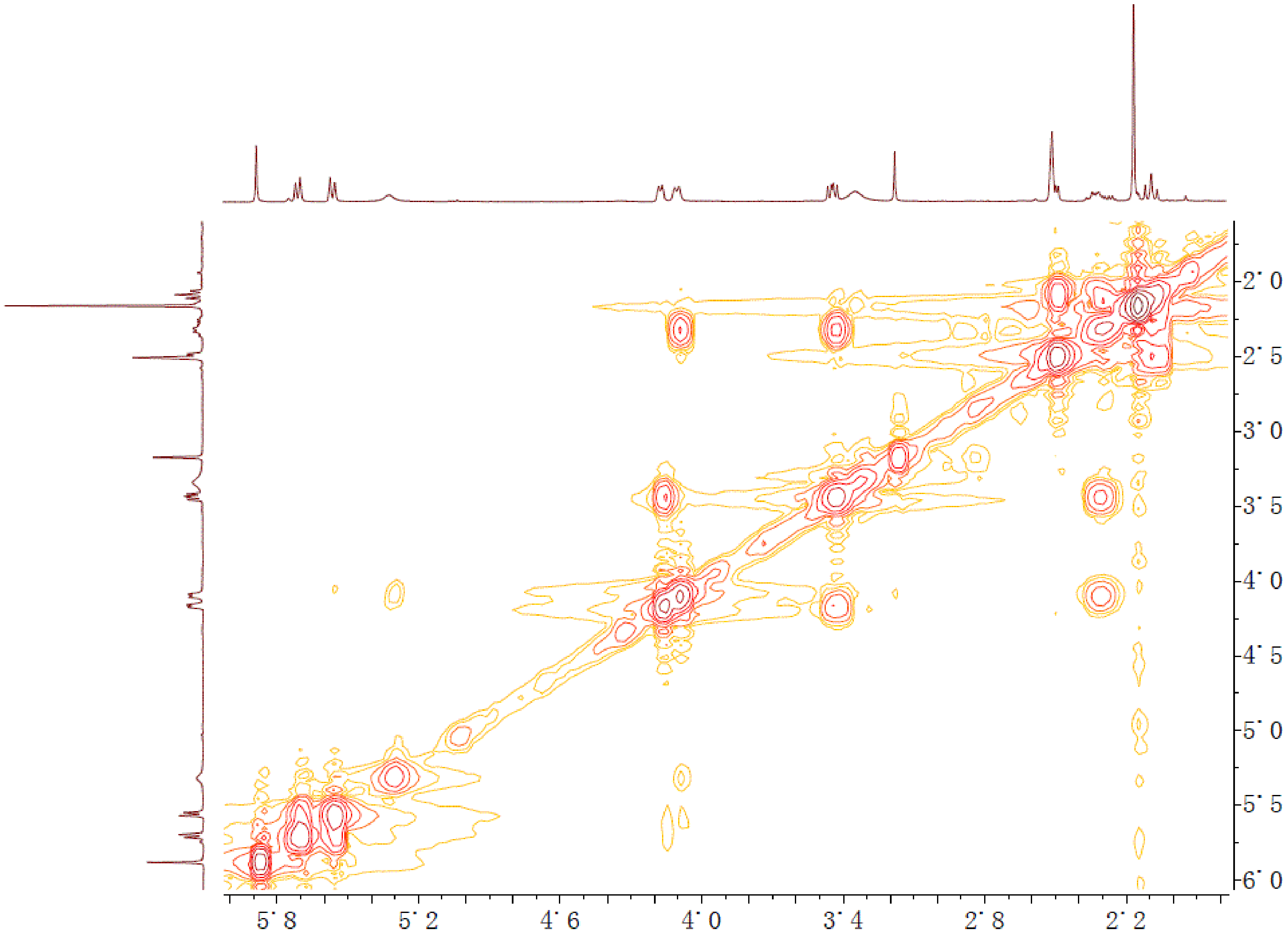

Figure S17. ^{1}H -^{1}H COSY spectrum of compound 3.

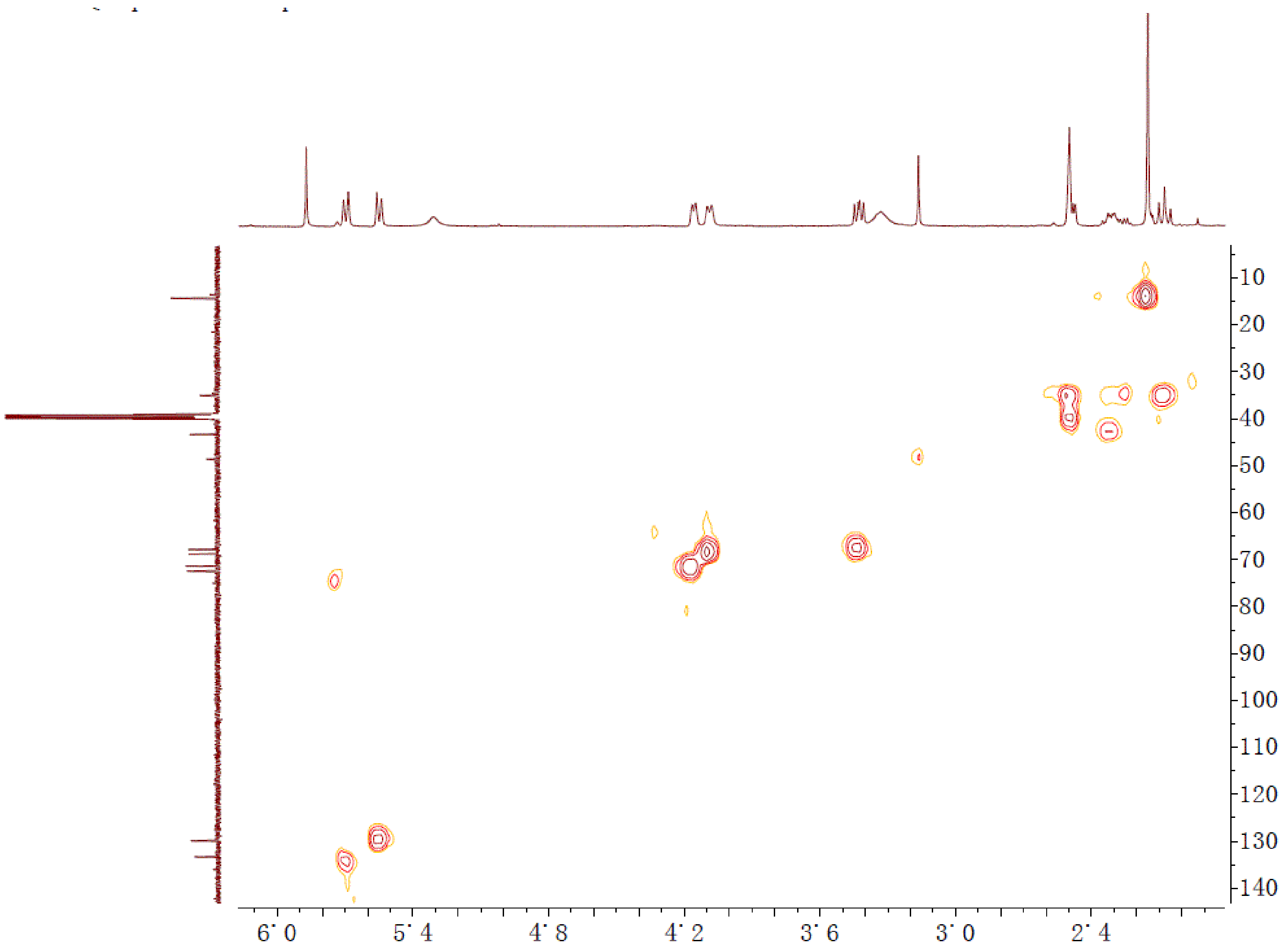

Figure S18. HSQC spectrum of compound **3**.

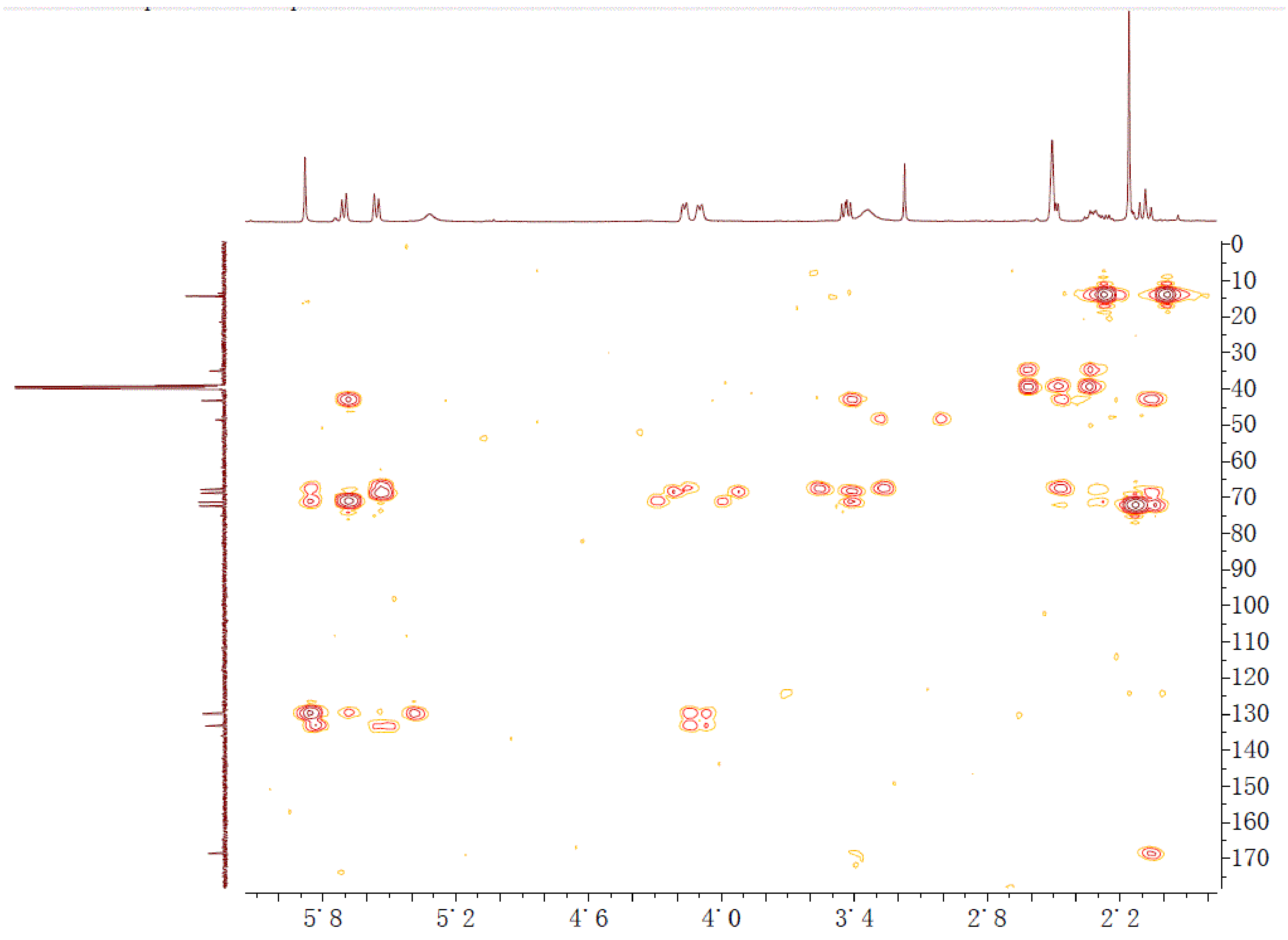

Figure S19. HMBC spectrum of compound **3**.

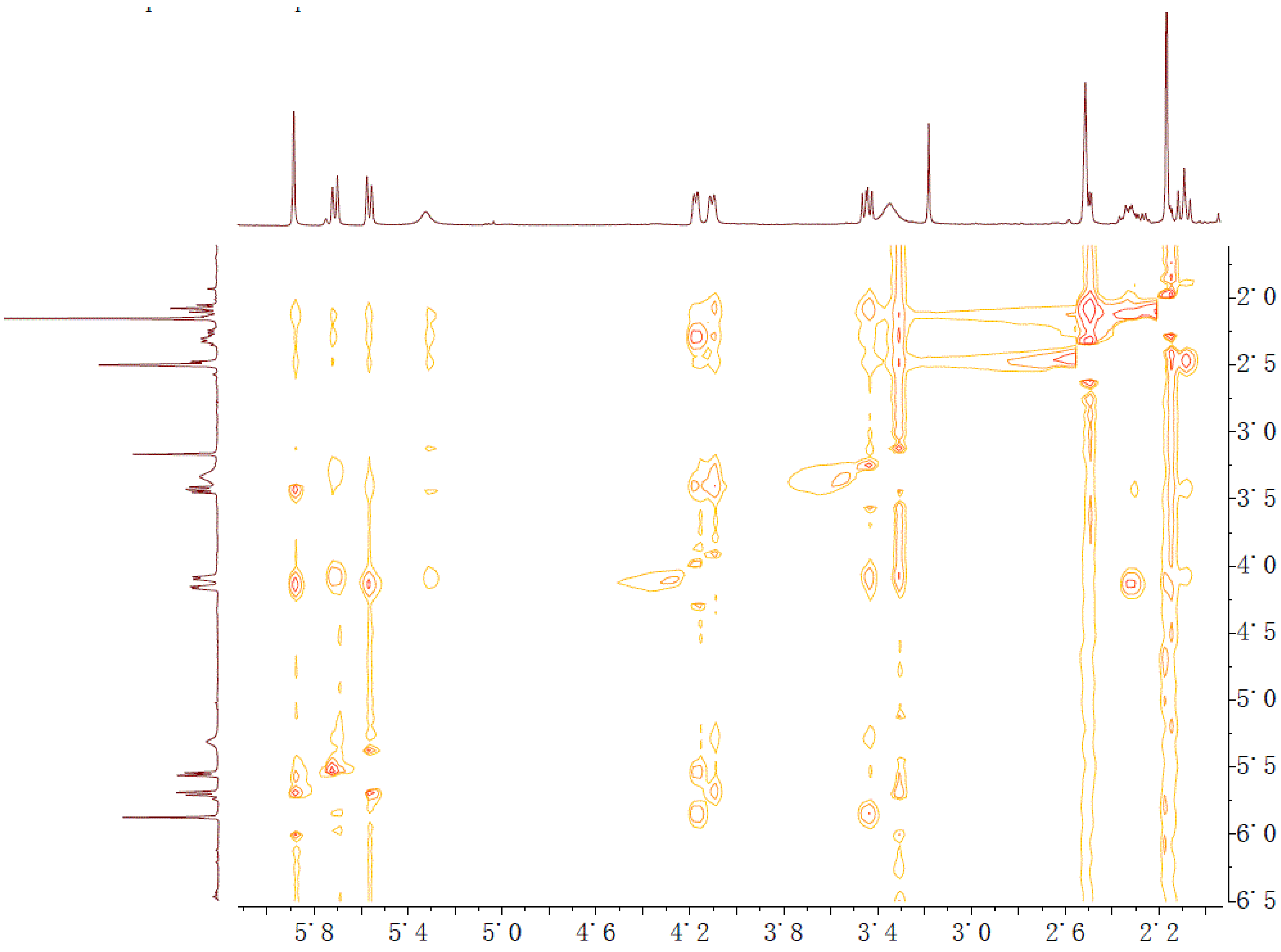

Figure S20. NOESY spectrum of compound **3**.

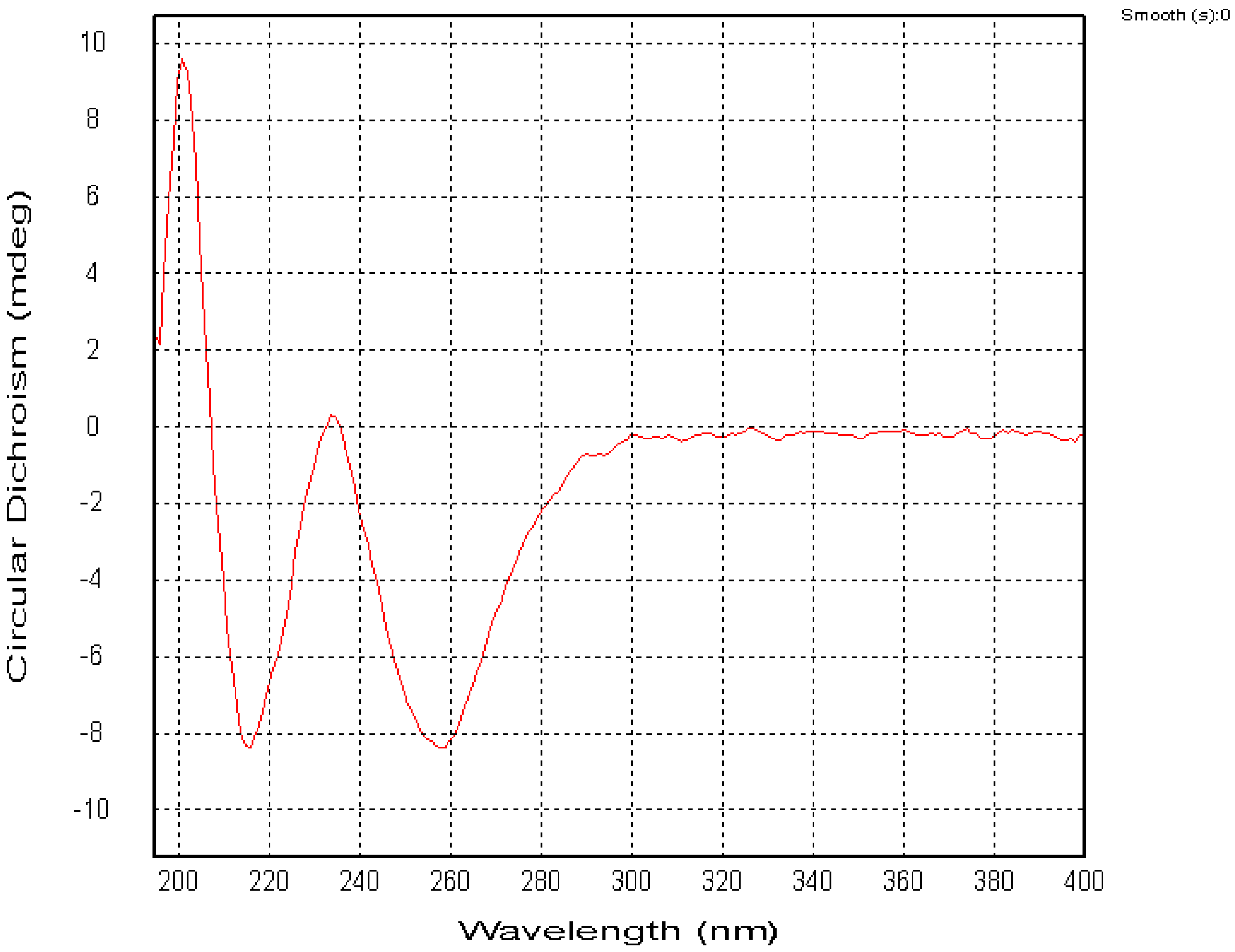

Figure S21. CD spectrum of compound **3**.

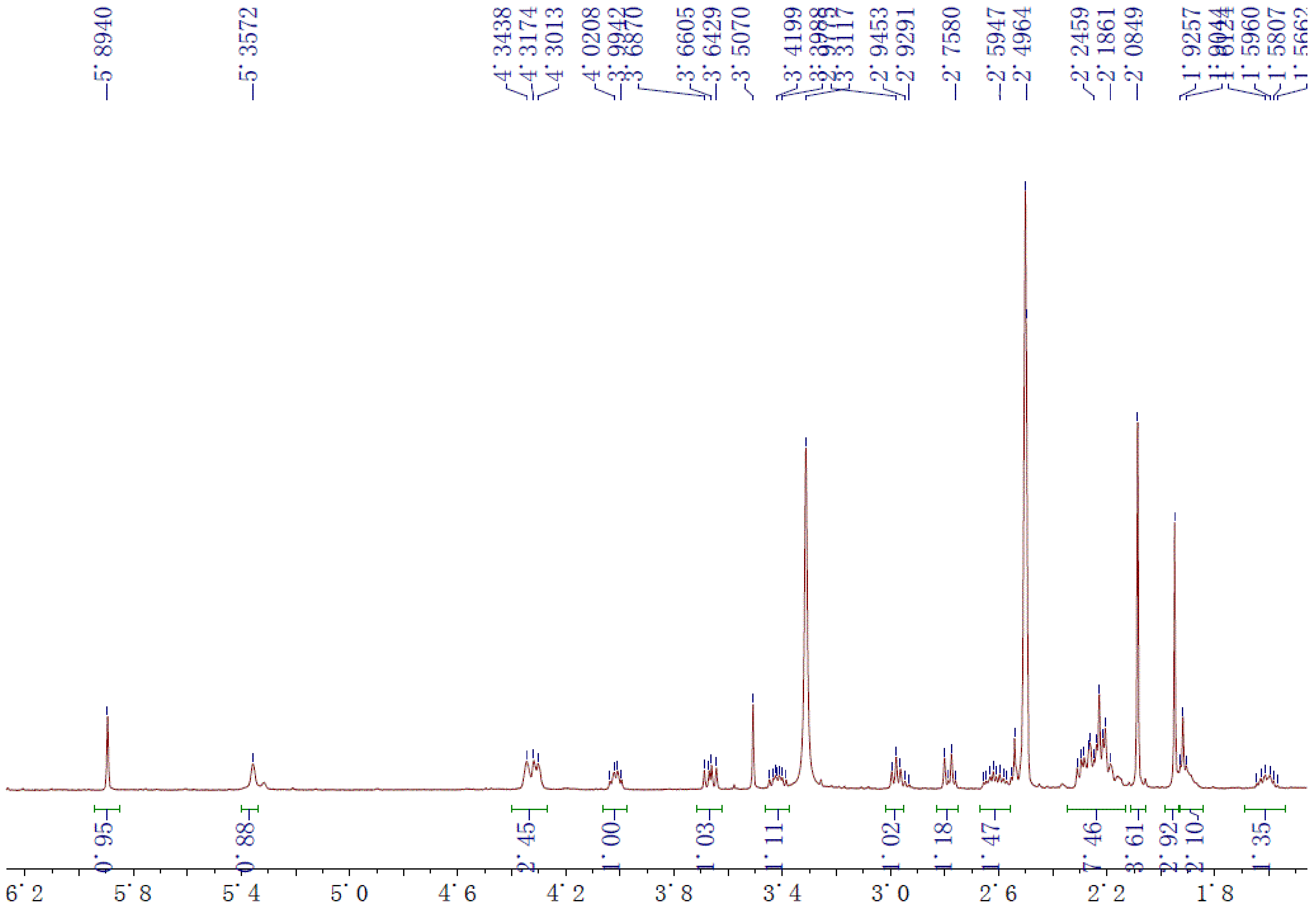

Figure S22. ^{1}H NMR (500 MHz, DMSO-d_6) spectrum of compound **4.**

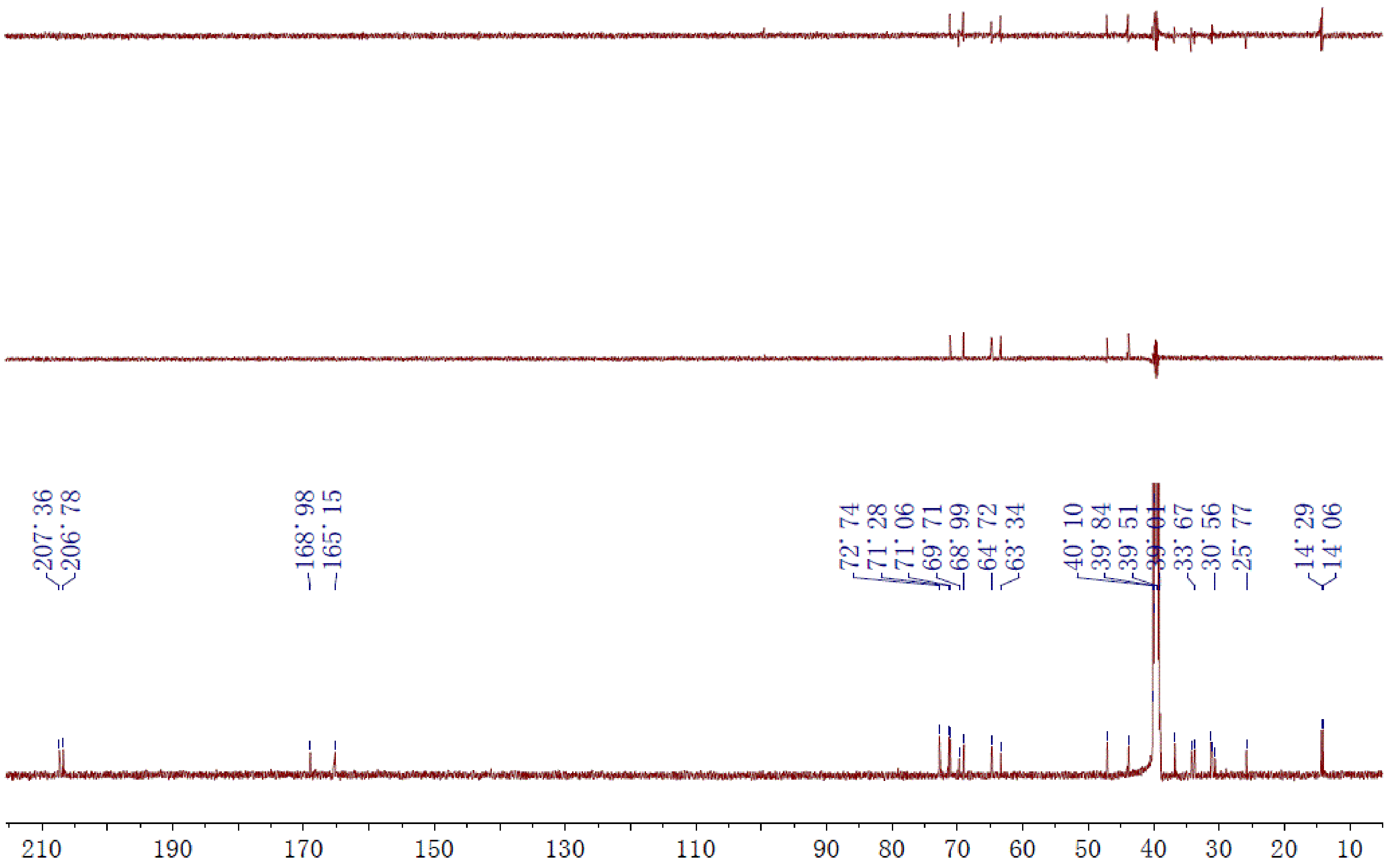

Figure S23. ^{13}C NMR (125 MHz, DMSO-d_6) and DEPT spectra of compound **4**.

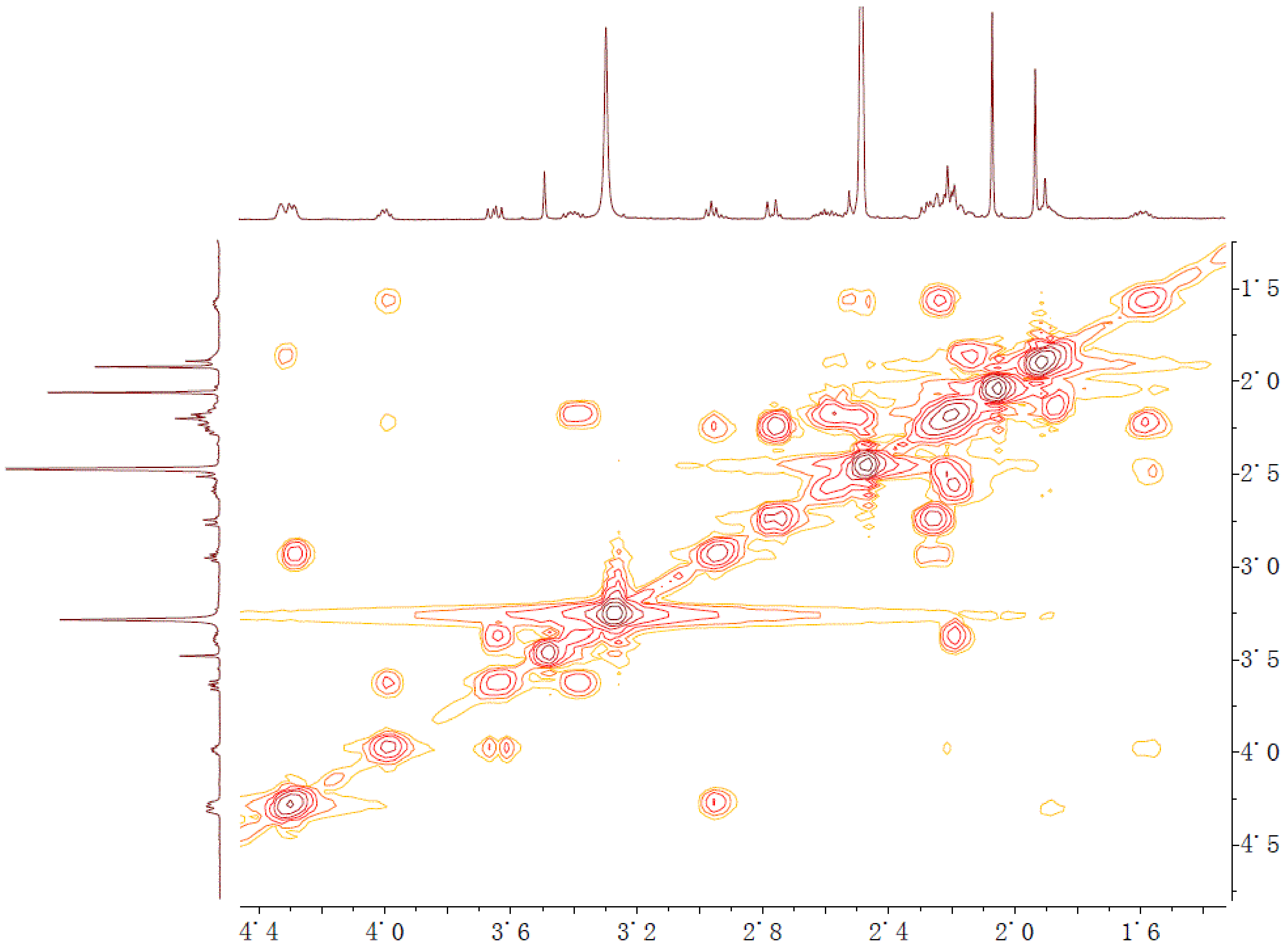

Figure S24. ^{1}H -^{1}H COSY spectrum of compound **4.**

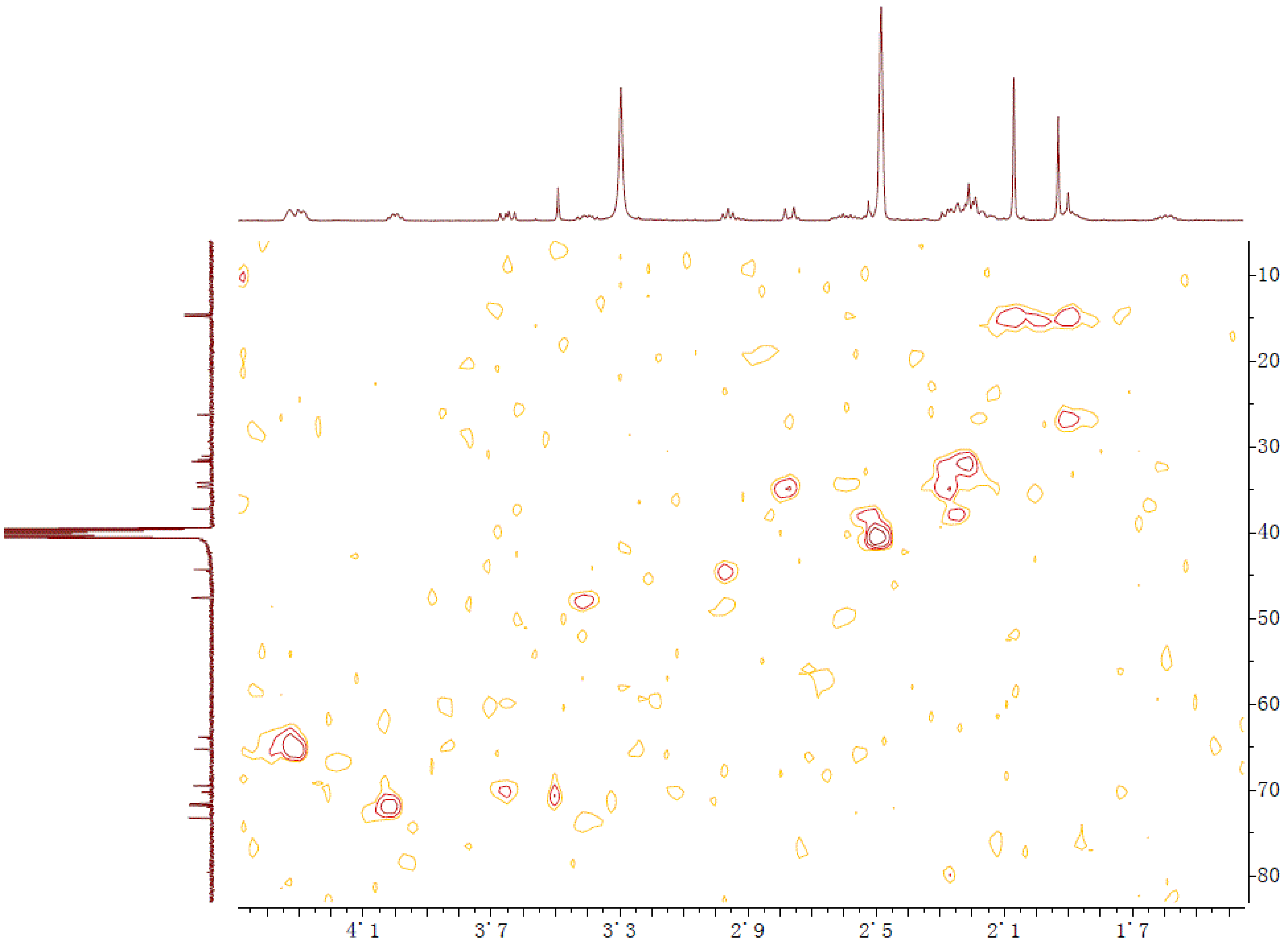

Figure S25. HSQC spectrum of compound **4**.

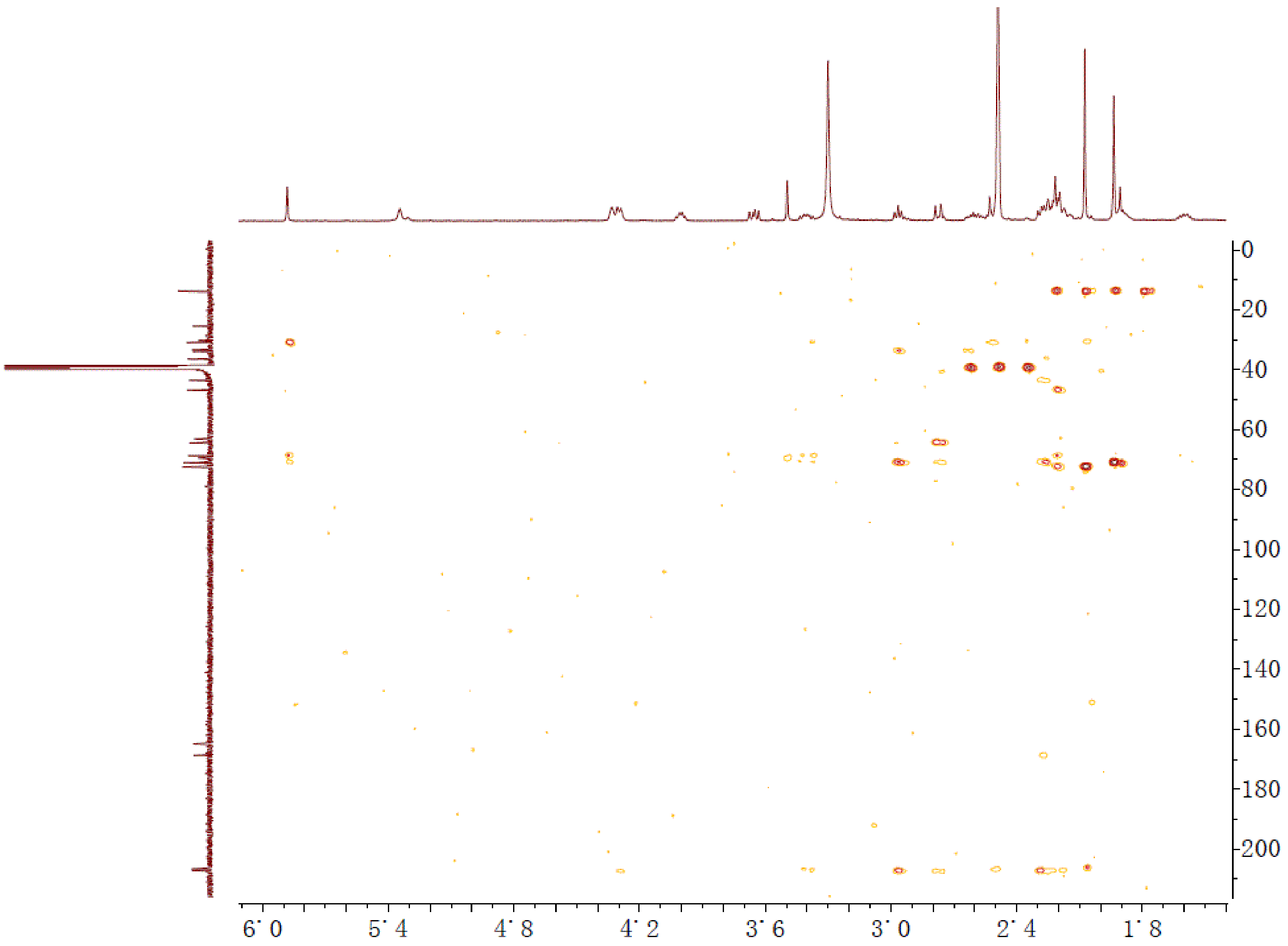

Figure S26. HMBC spectrum of compound **4**.

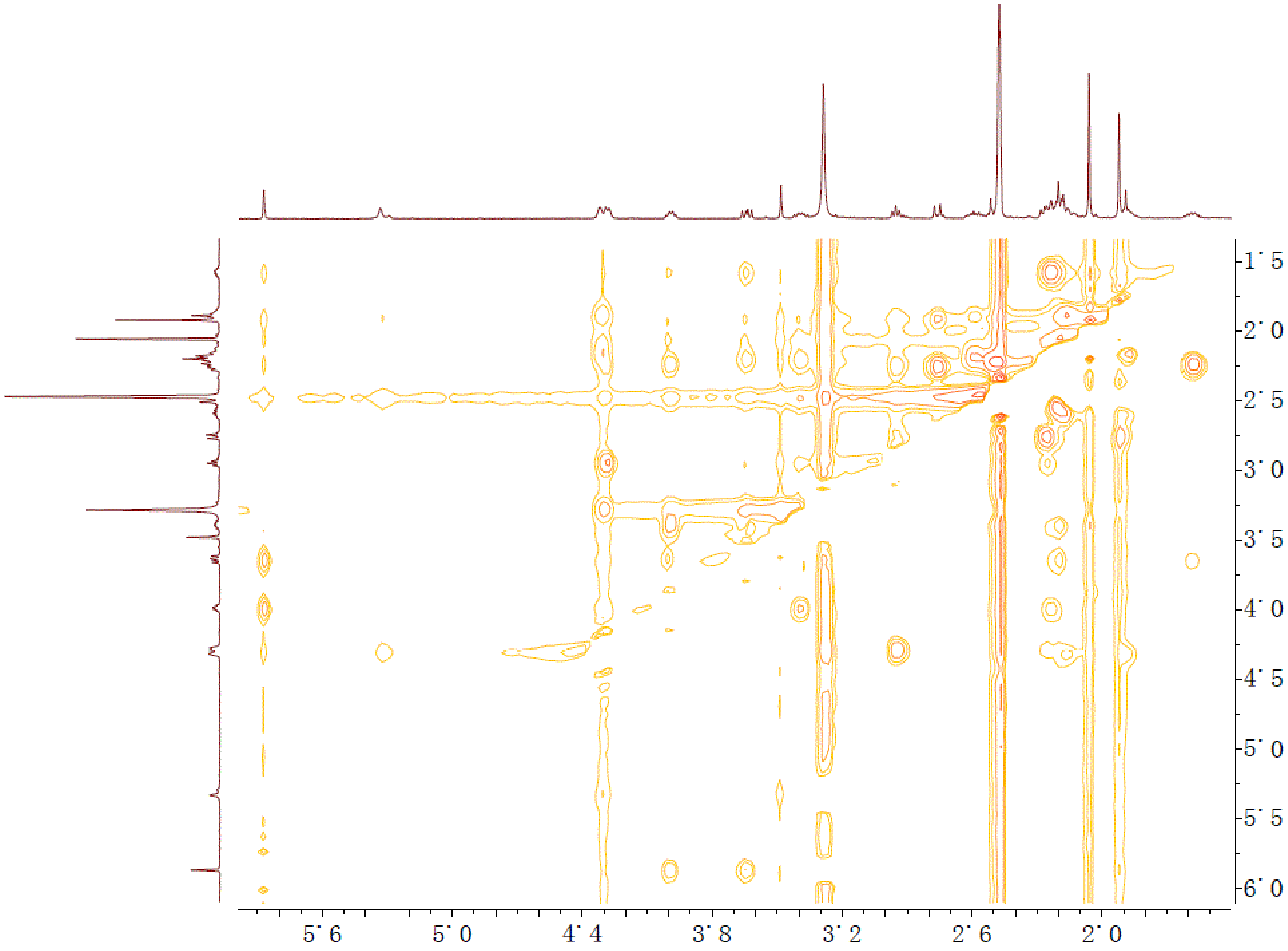

Figure S27. NOESY spectrum of compound **4**.

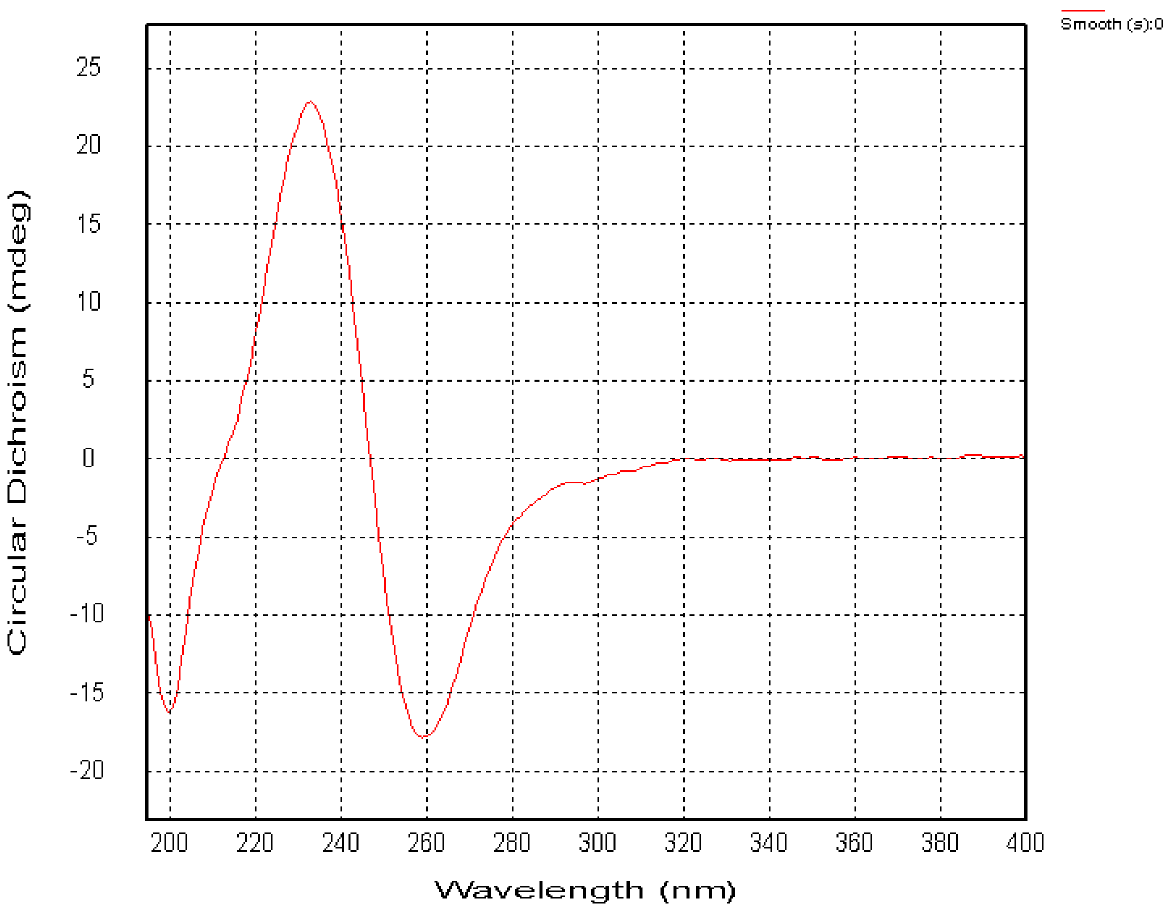

Figure S28. CD spectrum of compound **4**.

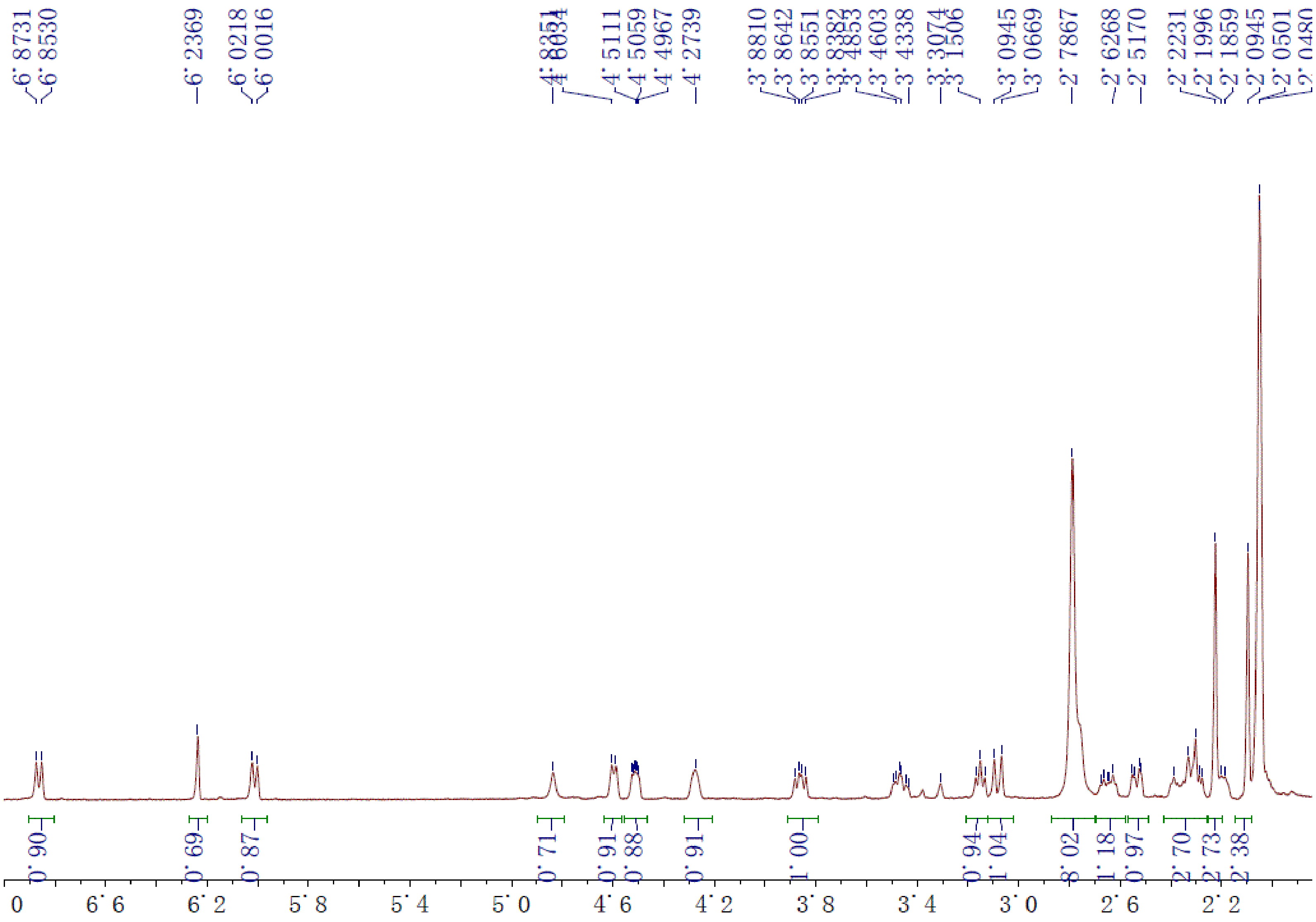

Figure S29. ^{1}H NMR (500 MHz, Acetone-d_6) spectrum of compound **5**.

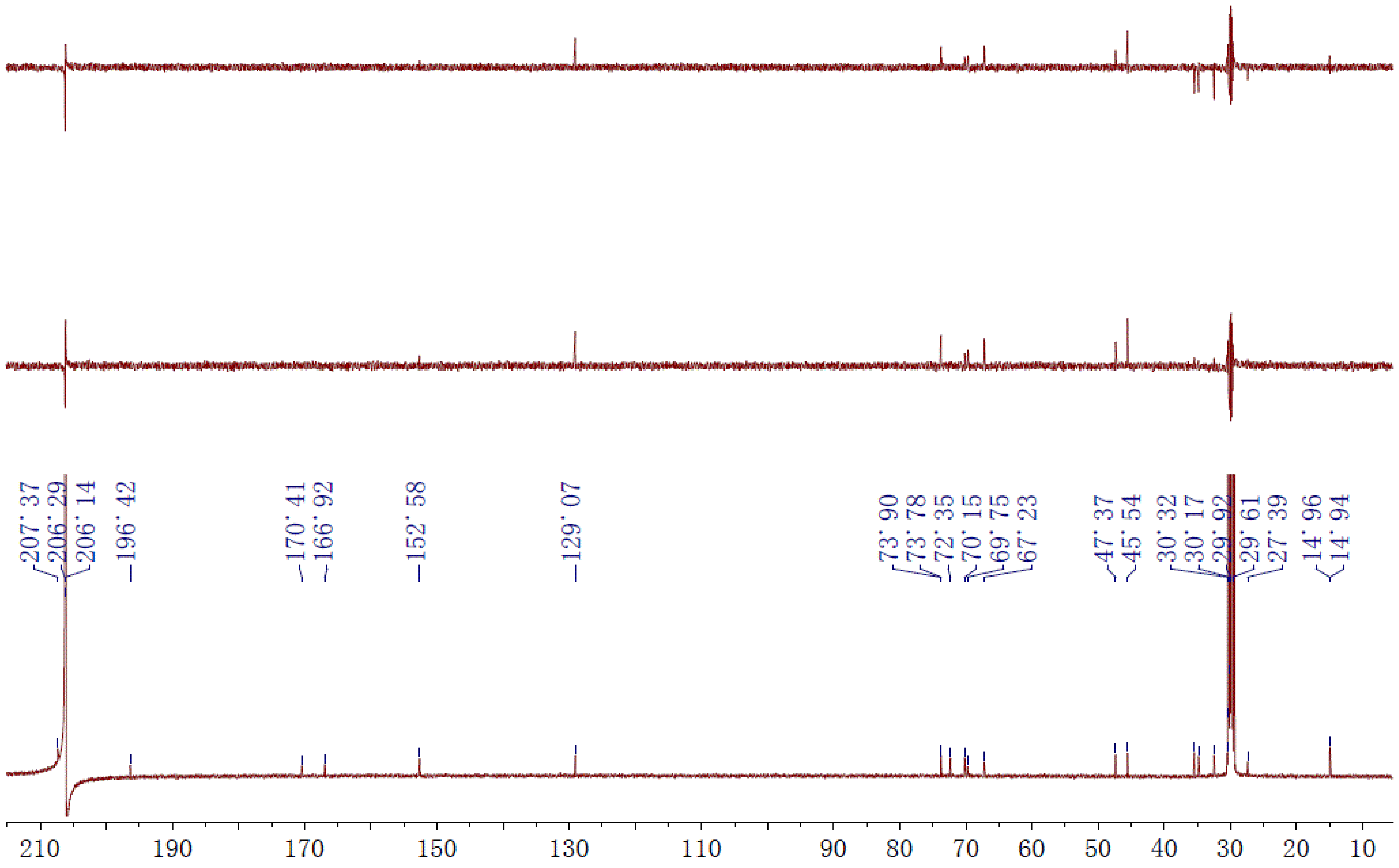

Figure S30. ^{13}C NMR (125 MHz, Acetone-d_6) and DEPT spectra of compound **5**.

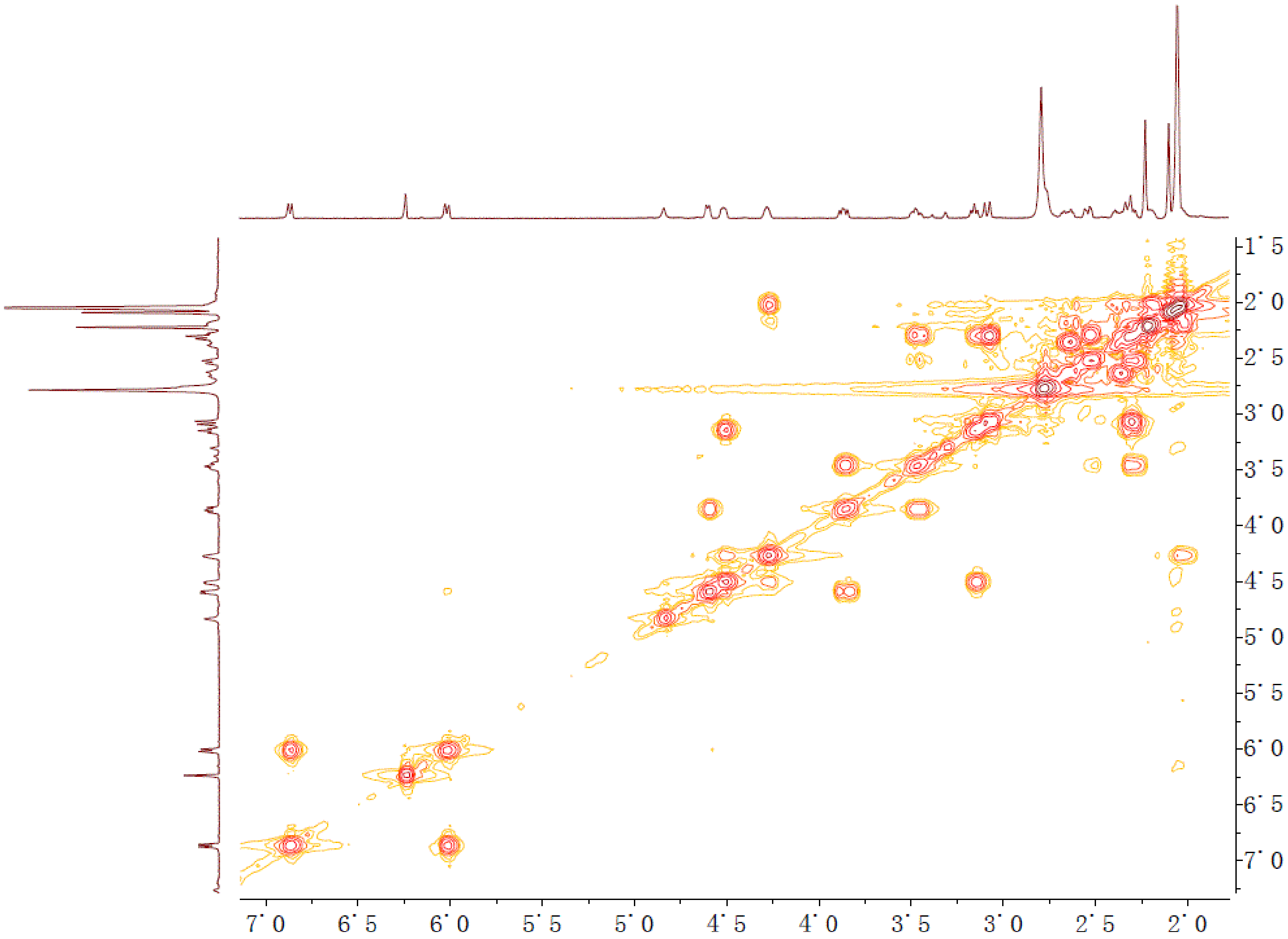

Figure S31. ^{1}H -^{1}H COSY spectrum of compound **5**.

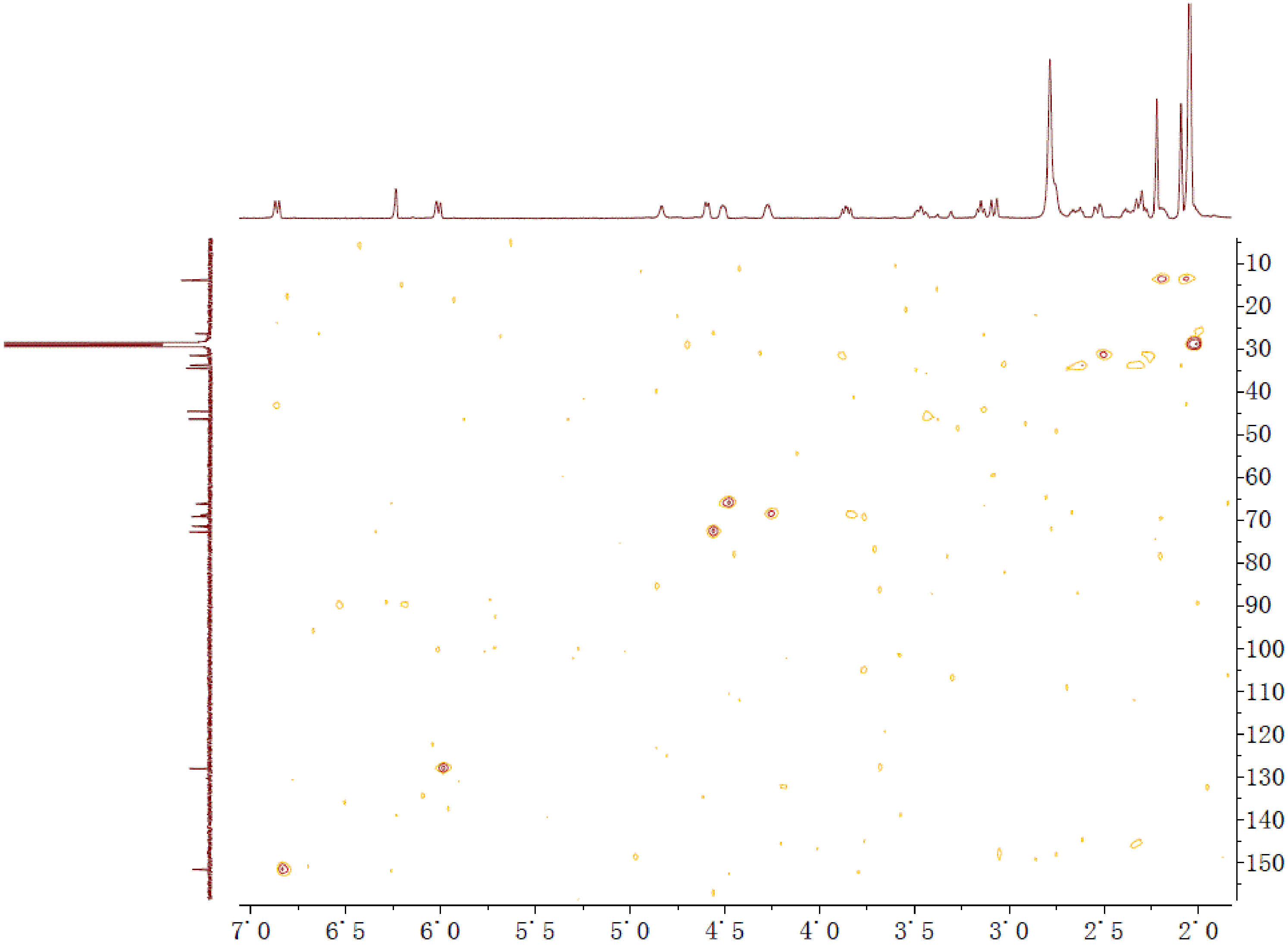

Figure S32. HSQC spectrum of compound **5**.

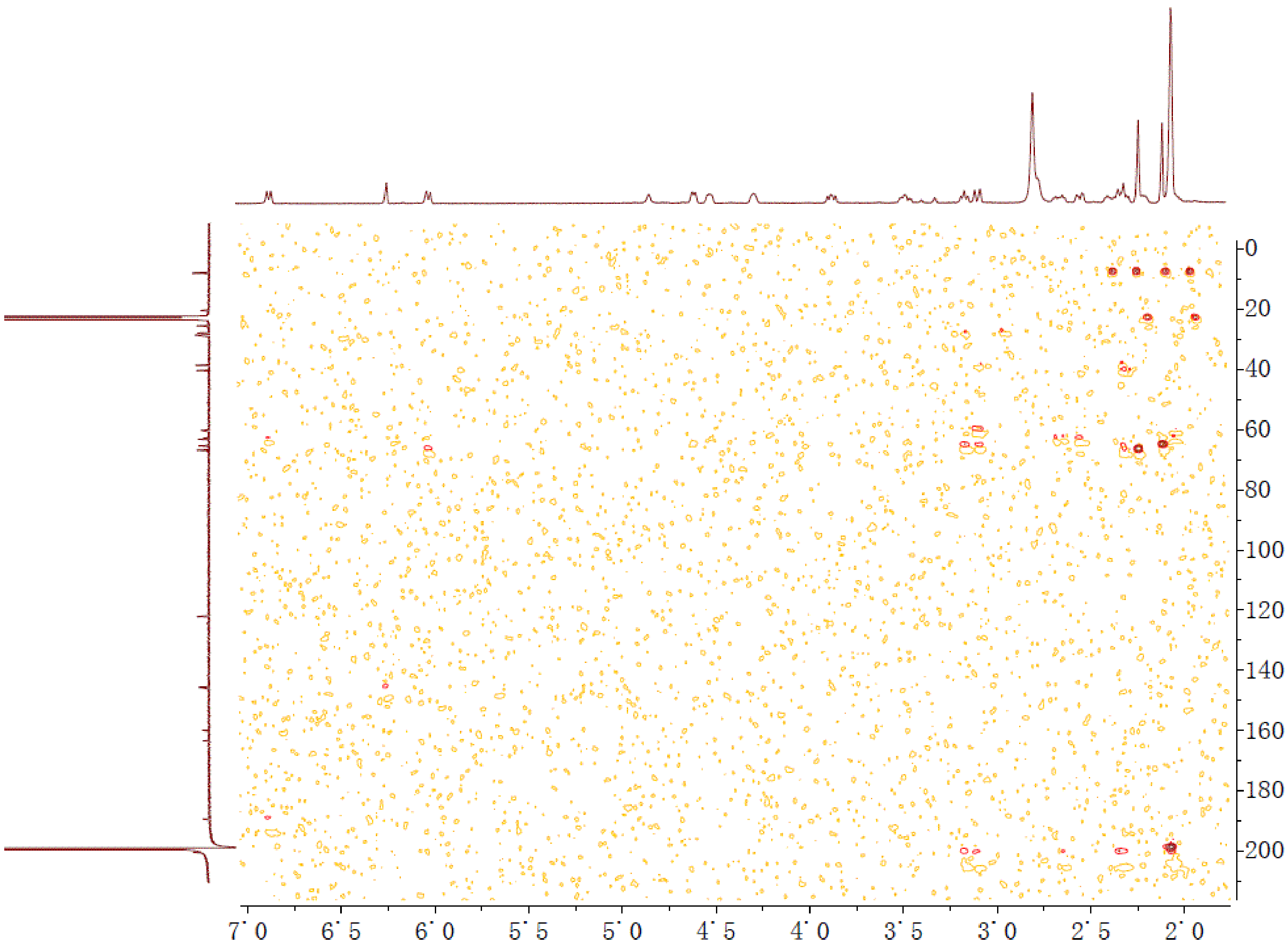

Figure S33. HMBC spectrum of compound **5**.

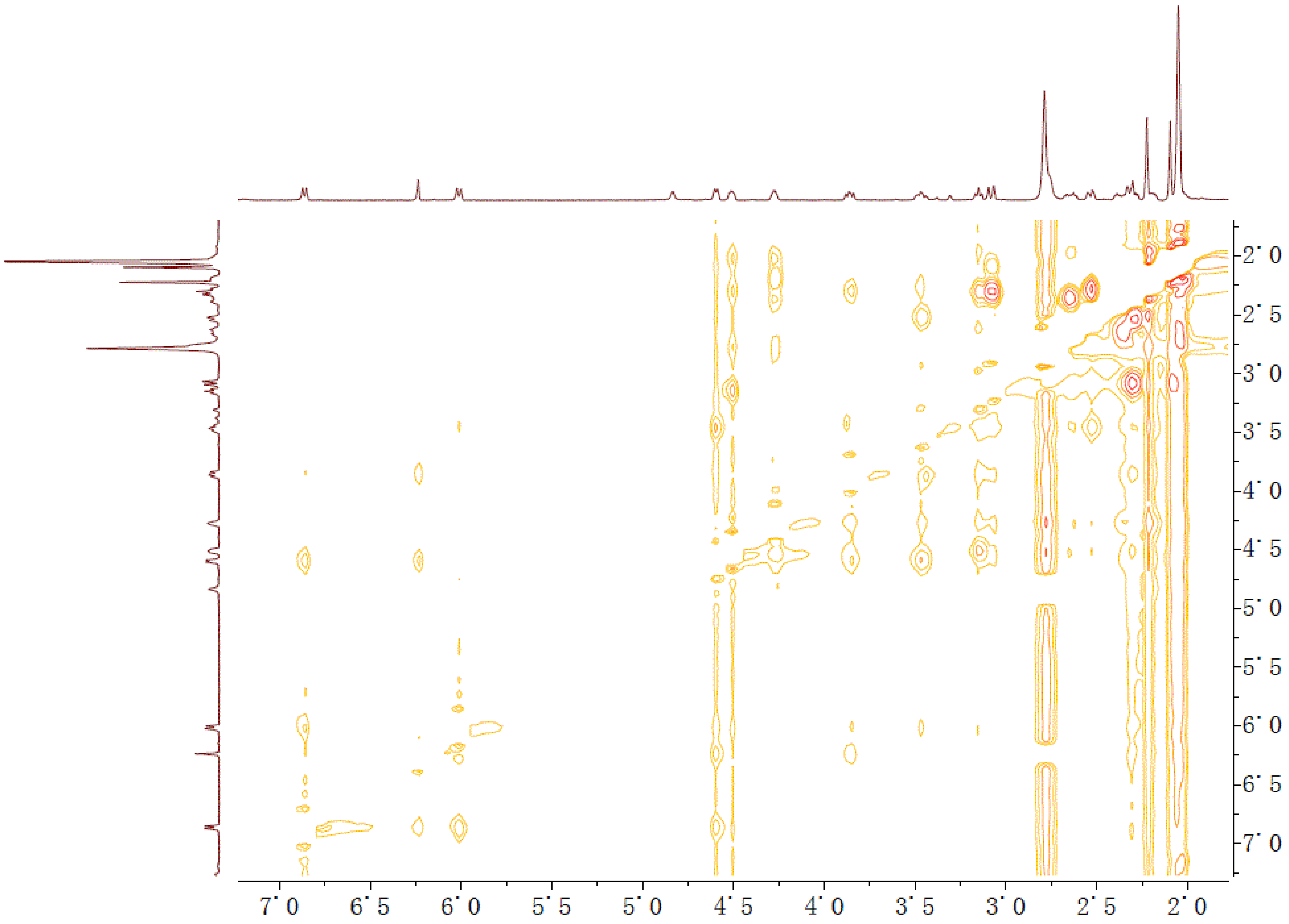

Figure S34. NOESY spectrum of compound **5**.

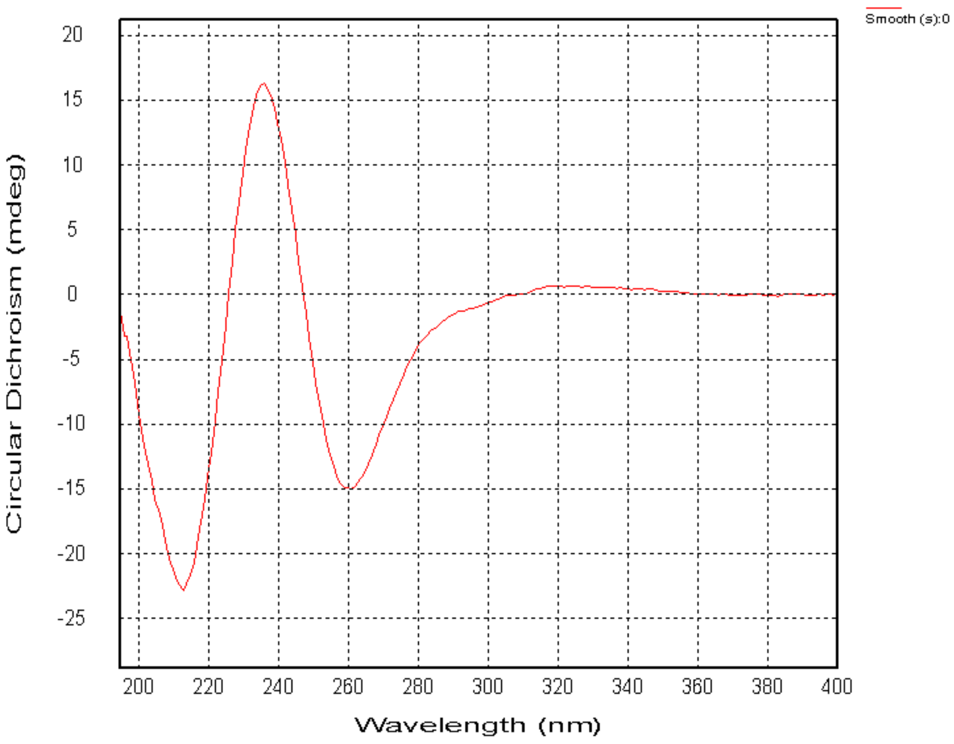

Figure S35. CD spectrum of compound **5**.

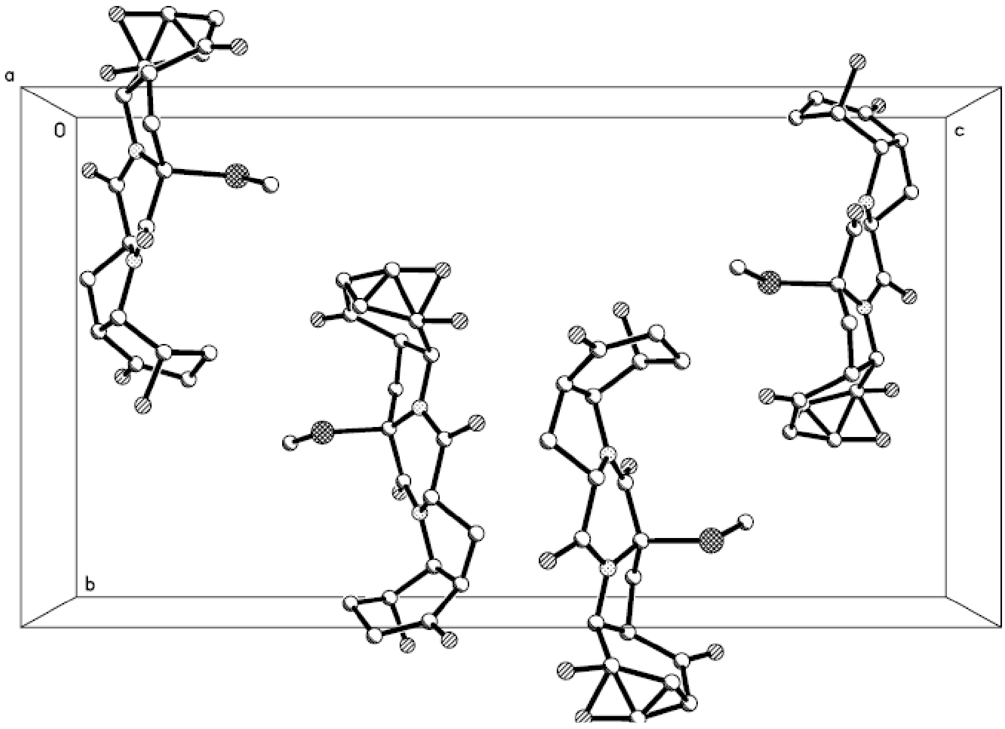

Figure S36. Crystal packing of compound **1** at 293(2) K.

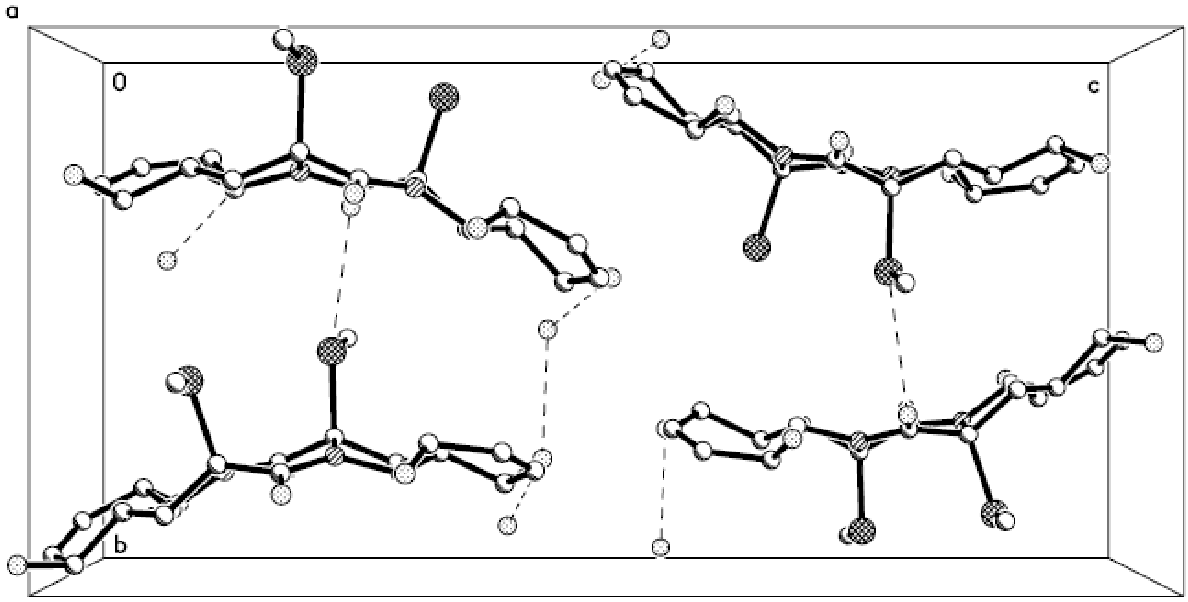

Figure S37. Crystal packing of compound **3** at 293(2) K.

ACCTCCCACCCGTGTTTACCGTACCCTGTTGCTTCGGCGCGCCCGCCCTCGCGGCCGCCGGGGGGCCTCCGCCCCCGGGCCCGTGCCCGCC
GAAGACACCCCTGAACGCTGTCTGAAGATTGTCGTCTGAGCGAATAGCGAAAAATAAGTTAAAACTTTCAACAACGGATCTCTTGGTTCC
GGCATCGATGAAGAACGCAGCGAAATGCGATAACTAATGTGAATTGCAGAATTCAGTGAATCATCGAGTCTTTGAACGCACATTGCGCCC
CCTGGTATTCCGGGGGGCATGCCTGTCCGAGCGTCATTGCTGCCCTCAAGCCCGGCTTGTGTGTTGGGCCGCCGTCCCCCCCTTCCCGGGG
GGACGGGCCCGAAAGGCAGCGGCGGCACCGCGTCCGGTCCTCGAGCGTATGGGGCTCTGTCACCCGCTCTGCAGGCCCGGCCGGCGCCA
GCCGACCCCCATCATCCTTTTTTTCAGGTTGACCTCGGATCA

Figure S38. ITS sequence of MA231.

© 2015 by the authors; licensee MDPI, Basel, Switzerland. This article is an open access article distributed under the terms and conditions of the Creative Commons Attribution license (http://creativecommons.org/licenses/by/4.0/).
